# Truxene-Centered Electron Acceptors for Non-Fullerene Solar Cells: Alkyl Chain and Branched Arm Engineering

**DOI:** 10.3390/ijms231810402

**Published:** 2022-09-08

**Authors:** Kaiwen Lin, Wenhao Du, Shuqi Shen, Haoshen Liang, Xiaobin Zhang, Manjun Xiao, Yuehui Wang

**Affiliations:** 1 Department of Materials and Food, University of Electronic Science and Technology of China Zhongshan Institute, Zhongshan 528402, China; 2 School of Optoelectronic Science and Engineering, University of Electronic Science and Technology of China, Chengdu 610054, China; 3 Key Lab of Environment-Friendly Chemistry and Application (Ministry of Education), College of Chemistry, Xiangtan University, Xiangtan 411105, China

**Keywords:** non-fullerene solar cells, electron acceptors, star-shaped, truxene, 3-ethylrhodanine

## Abstract

A series of symmetrical truxene-centered and 3-ethylrhodanine end-capped electron acceptors with high absorption coefficient, namely Tr(Hex)_6_-3RD, Tr(Dec)_6_-3RD, and Tr(Hex)_6_-6RD, were prepared and constructed for non-fullerene solar cells. To satisfy solution-processability, multiple energy levels, and suitable morphology, these three acceptors were comparatively studied through alkyl chain (hexyl/decyl) and branched-arm engineering (three/six branched arms). The six-bladed propeller acceptor of Tr(Hex)_6_-6RD recorded the power conversion efficiency (PCE) of 1.1% blending with PTB7-Th without additional additives and post-processing. This work highly broadens the potential applications of star-shaped truxene building blocks in the fields of organic electronics.

## 1. Introduction

Fullerene organic solar cells, whose active layers consists of electron donors and fullerene derivative acceptors, have drawn a lot of attention and made significant development. Due to fullerene derivatives’ strong electron affinity and mobility, fullerene-based organic solar cells exhibited power conversion efficiencies (PCEs) surpassed 11% along with effective exciton dissociation and charge transport [1,2]. However, fullerene derivative acceptors have sums of disadvantages, including expensive cost, low visual absorption, poor photochemical stability, and limited energy level flexibility [3,4]. Recently, non-fullerene small molecular acceptors (SMAs) have become alternative promising candidates and made significant progress due to their easy tunable energy levels, outstanding chemical stability and photostability, as well as beneficial compatibility with donors to produce acceptable morphology [5,6]. The recorded high PCEs can be attributed to highly efficient molecular design strategies such as main chain engineering and side chain modification, which tune the spectral features, electronic energy levels, molecular aggregation, mobility, and blend morphology [7,8]. Therefore, developing new types of high-performance SMAs is still a huge challenge and is of great importance.

It is well known that broad absorption and high absorption coefficients enable the active layer to receive more photons [4,5,6,8]. Naturally, well-matched donors and acceptors would be helpful to achieve complementary absorption and further improve the PCEs’ performance. However, materials with high absorption coefficients do not come by easily. State-of-the-art SMAs, ITIC, and Y6 display outstanding light harvesting properties with high absorption coefficients [9,10]. The higher the absorption coefficient, the more photons are involved in achieving high PCEs. According to this instruction, multiple branched arms materials excite significant interest by combining the centers, π-conjugated bridge units, and terminal groups, since these materials are capable of harvesting incident light effectively owing to the stretch of branched arms in different directions. As the representative multiple branched arms materials, triphenylamine- and porphyrin-centered donors or acceptors have reported high molecular absorption coefficients exceeding 1.0 × 10^5^ cm^−1^ [11,12]. In addition, multiple branched arms materials can balance the aggregation and exciton diffusion to achieve satisfactory electronic communication [13,14]. Meanwhile, for multiple branched arms materials, a suitable planarization and rigidification center is necessary, which not only exhibits horizontal orientation to capture photons, but also enhances π-electron delocalization and accelerates charge transfer property [15].

The possibility of creating multiple branched arms truxene-centered SMAs is of importance here. Truxene was succeeded and widely used in optoelectronic fields in the last decade, including perovskite solar cells [16], dye-sensitized solar cells [17], two-photon absorption [18], photocatalysts [19], nonlinear optical (NLO) [20], organic light emitting diodes (OLEDs) [21], organic field effect transistors (OFETs) [22], and OSCs [23]. The truxene unit is regard as a nanosized rigid planar and C3-symmetric building block for generating numerous branching arms materials, which is capable of capturing as much incident light as feasible [24,25]. Moreover, truxene-centered SMAs are expected to perform strong intramolecular charge-transfer properties and consequently broad low-energy optical transitions, which is attributed to the well-conjugated structure and electron-rich property of truxene [26,27]. In addition, alkyl chain length and branched arm number changes also play crucial roles in controlling the intermolecular interaction, molecular packing, and charge-transport properties [28,29].

**Figure 1:**
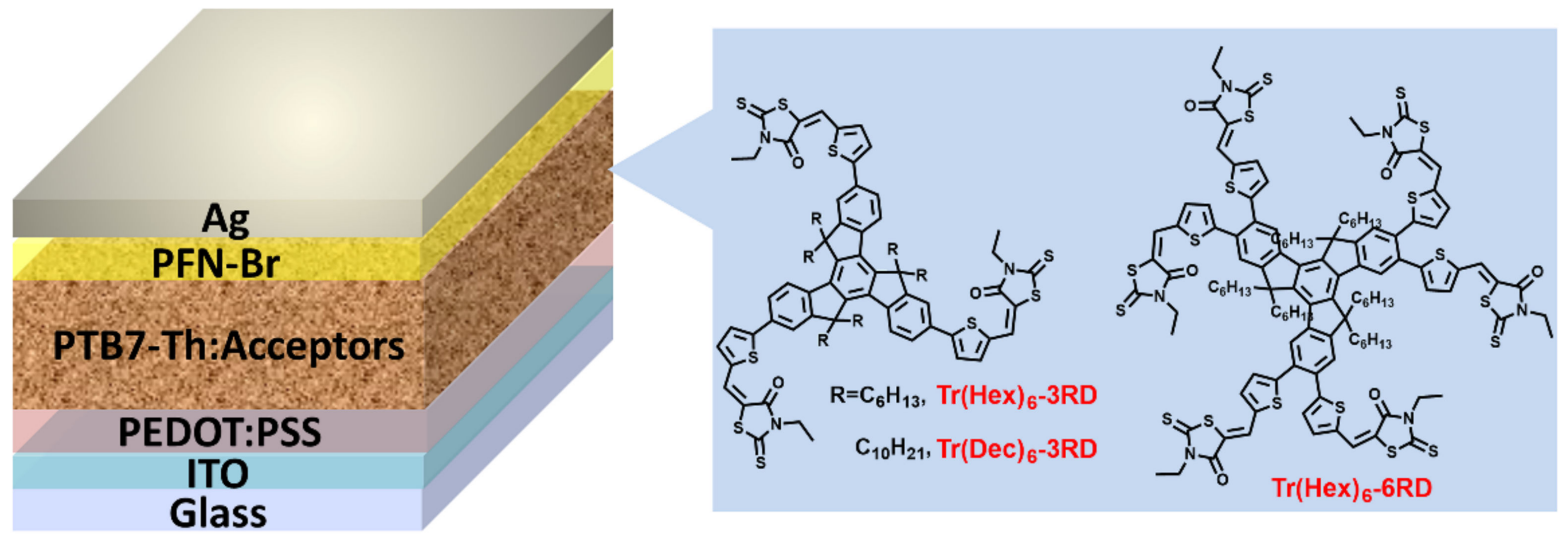
Device and chemical structures of truxene-centered acceptors.

On the basis of strategies mentioned above, herein we introduced three SMAs, namely (5E,5′E,5″E)-5,5′,5″-(((5,5,10,10,15,15-hexahexyl-10,15-dihydro-5H-diindeno[1,2-a:1′,2′-c]fluorene-2,7,12-triyl)tris(thiophene-5,2-diyl))tris(methaneylylidene))tris(3-ethyl-2-thioxothiazolidin-4-one) [Tr(Hex)_6_-3RD], (5E,5′E,5″E)-5,5′,5″-(((5,5,10,10,15,15-hexakis(decyl)-10,15-dihydro-5H-diindeno[1,2-a:1′,2′-c]fluorene-2,7,12-triyl)tris(thiophene-5,2-diyl))tris(methaneylylidene))tris(3-ethyl-2-thioxothiazolidin-4-one) [Tr(Dec)_6_-3RD], and (5Z,5′Z,5″Z,5′′′E,5′′′′E,5′′′′′E)-5,5′,5″,5′′′,5′′′′,5′′′′′-(((5,5,10,10,15,15-hexahexyl-10,15-dihydro-5H-diindeno[1,2-a:1′,2′-c]fluorene-2,3,7,8,12,13-hexayl)hexakis(thiophene-5,2-diyl))hexakis(methaneylylidene))hexakis(3-ethyl-2-thioxothiazolidin-4-one) [Tr(Hex)_6_-6RD], of which a truxene unit is chosen as the center electron donor, thiophene as the bridge unit, and 3-ethylrhodanine as the terminal electron acceptor (Figure 1). These SMAs were further utilized in OSCs devices. The symmetric truxene-centered SMAs exhibit not only good solution-processability and thermal stability but also the adjustment of suitable blend morphology, which have been comparatively discussed from structure –property relationship in this research.

## 2. Results and Discussion

**Scheme 1:**
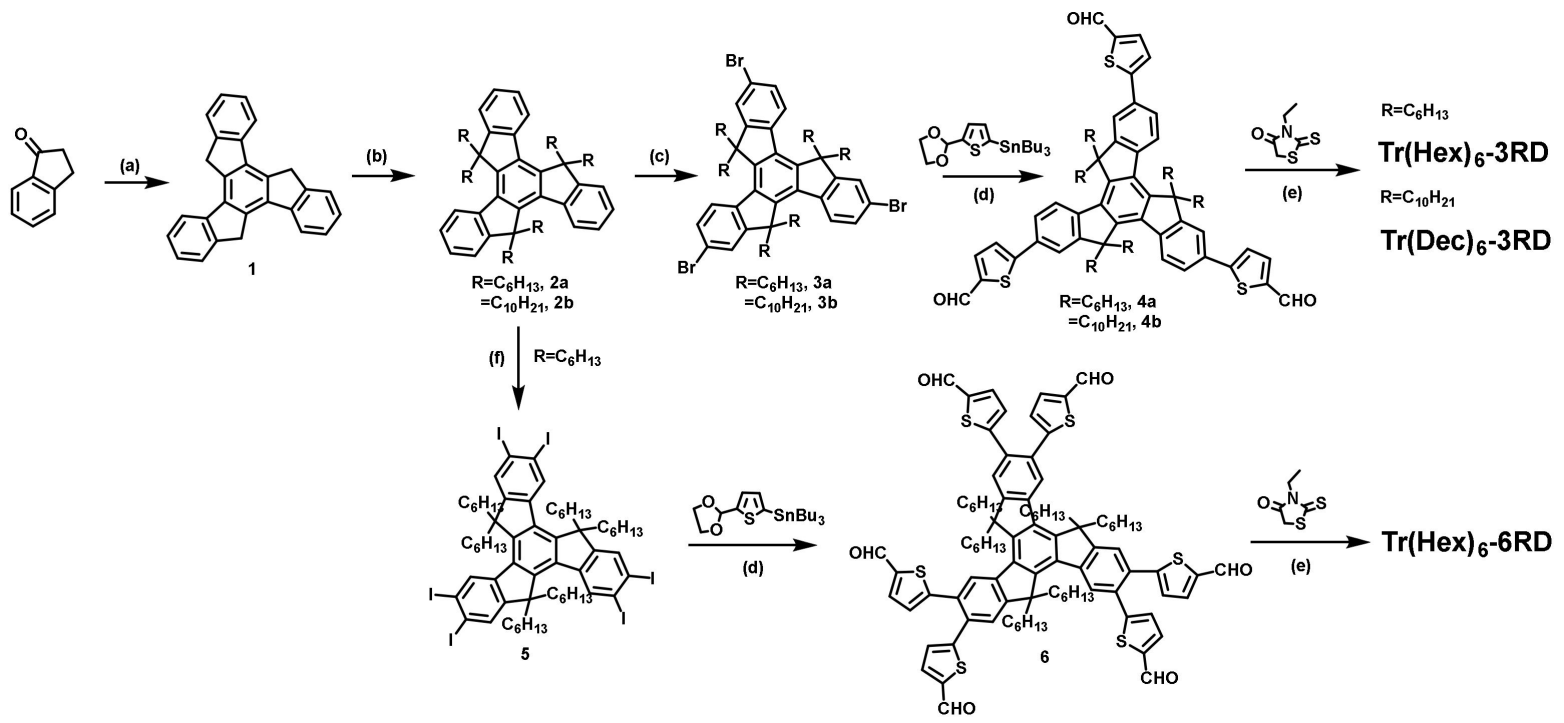
Synthetic routes of truxene-centered SMAs. Reagents and conditions: (a) HCl, HOAc, 120 °C; (b) *n*-BuLi, THF, −78 °C; 1-bromohexene/1-bromodecane; (c) FeCl_3_, CHCl_3_, Br_2_, 0 °C; (d) Pd(PPh_3_)_4_, toluene, 100 °C; (e) Chloroform, pyridine/piperidine, 65 °C; (f) [bis(trifluoroacetoxy)iodo]benzene, I_2_, DCM, RT.

**Synthesis and characterization** The molecular design and synthesis of truxene-centered SMAs is of great importance because it exhibited the possibility as a broad-band gap acceptors in OSCs. The precise synthesis processes are discussed in the Materials and Methods section. Scheme 1 depicts the synthetic routes such as truxene generation, bromization/iodine reaction, Stille cross-coupling reaction, and Knoevenagel condensation to afford truxene-centered SMAs. The structures of all intermediates and final products were confirmed by ^1^H NMR and ^13^C NMR (Figures S1–S18). Moreover, in terms of guaranteeing the solubility and the resulting promoting the solution processability, alkyl chain length and branched arm number changes are employed. According to the design concept, all these acceptors are dissolved readily in common organic solvents at room temperature, ensuring the preparation of the smooth and uniform films in OSCs devices. The solubility of truxene-centered SMAs in chloroform was as follows (Table S1): Tr(Hex)_6_-3RD ~80, Tr(Dec)_6_-3RD ~130, and Tr(Hex)_6_-6RD ~180 mg mL^−1^, respectively. Truxene-centered SMAs modified by decyl chain instead of hexyl chain exhibited better solubility. Six branched arms acceptors of Tr(Hex)_6_-6RD showed the best solubility as expected. 

**Figure 2:**
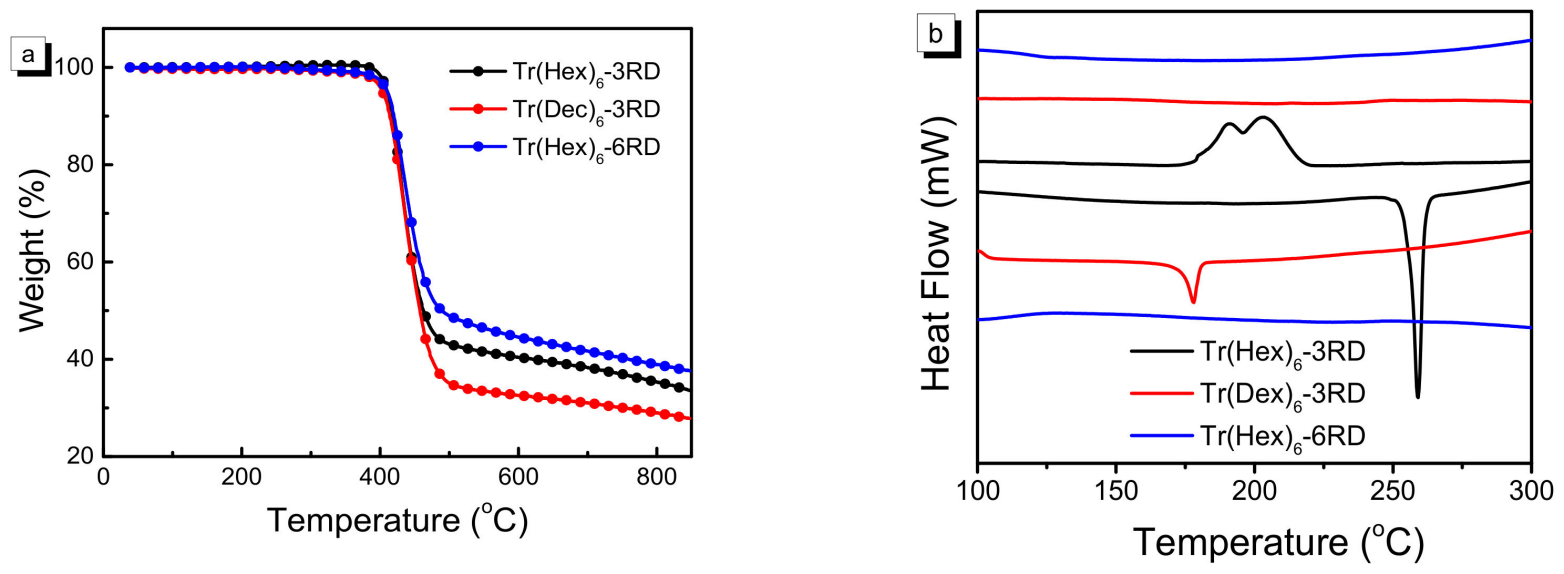
(**a**) TGA and (**b**) DSC spectra of truxene-centered acceptors.

Under a nitrogen environment, the thermal characteristics of truxene-centered SMAs were investigated using thermogravimetric analysis (TGA) and differential scanning calorimetry (DSC), and the results are presented in Figure 2. All truxene-centered SMAs exhibited outstanding thermal stability with 5% weight loss above 400 °C, which was in accordance with the report research (Figure 2a) [30]. Tr(Hex)_6_-3RD displayed obvious melting (*T*_m_ = 259 °C) and double crystallization peaks (*T*_c_ = 203/190 °C). Tr(Dec)_6_-3RD only showed weak melting peak of 178 °C. Tr(Hex)_6_-6RD, on the other hand, presented no apparent phase transition, indicating its amorphous nature (Figure 2b).

**Figure 3:**
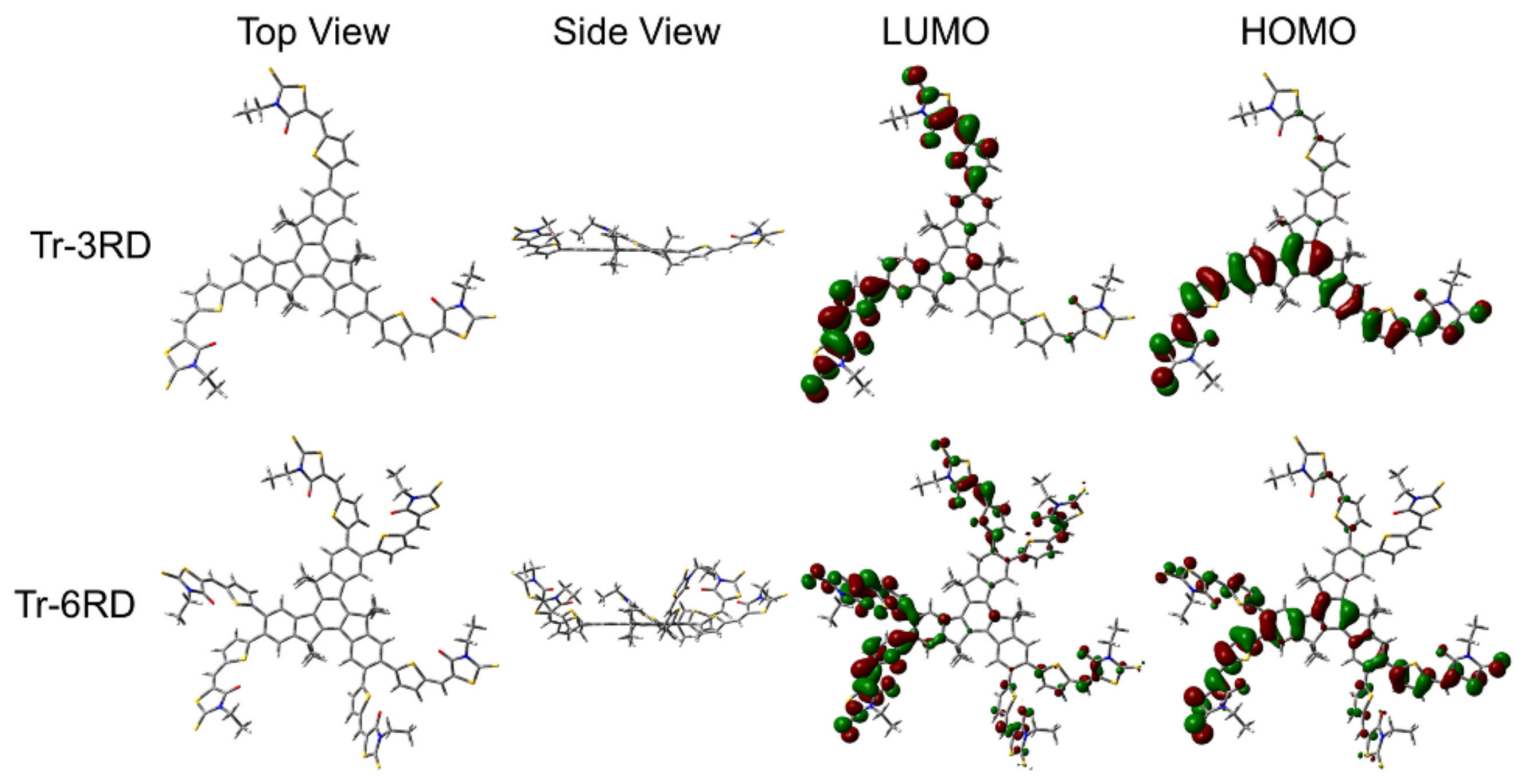
DFT computed molecular geometries and molecular frontier orbital for the model structures (Tr-3RD and Tr-6RD).

**Theoretical calculations** Density functional theoretical (DFT) calculations were carried out at the B3LYP/6-31G(d) level to optimize the geometries and molecular frontier orbital so as to better study these truxene-centered SMAs [31]. Methyl group was introduced instead of hexyl and decyl chain on truxene to facilitate the calculation, and the model structures were given the names of Tr-3RD and Tr-6RD. As shown in Figure 3, both model structures were twisted like three- or six-bladed propellers. The three branched arms structure of Tr-3RD displayed dihedral angles of 23° between truxene and adjacent thiophene units. As the number of branched arms increased to six, the truxene unit and the branched arms tightly twisted with an angle of 59° between the truxene and thiophene unit, owing to the strong steric hindrance, indicating that the increase of branched arms leads to a more distorted propeller configuration.

The molecular frontier orbital of Tr-3RD and Tr-6RD were also estimated. Tr-6RD exhibited deeper highest occupied molecular orbitals (HOMO, −5.71 eV) and lowest unoccupied molecular orbitals (LUMO, −2.86 eV) levels in comparison with Tr-3RD (HOMO/LUMO, 5.55/−2.74 eV), which coincide with the aforementioned cyclic voltammetry measurement.

**Figure 4:**
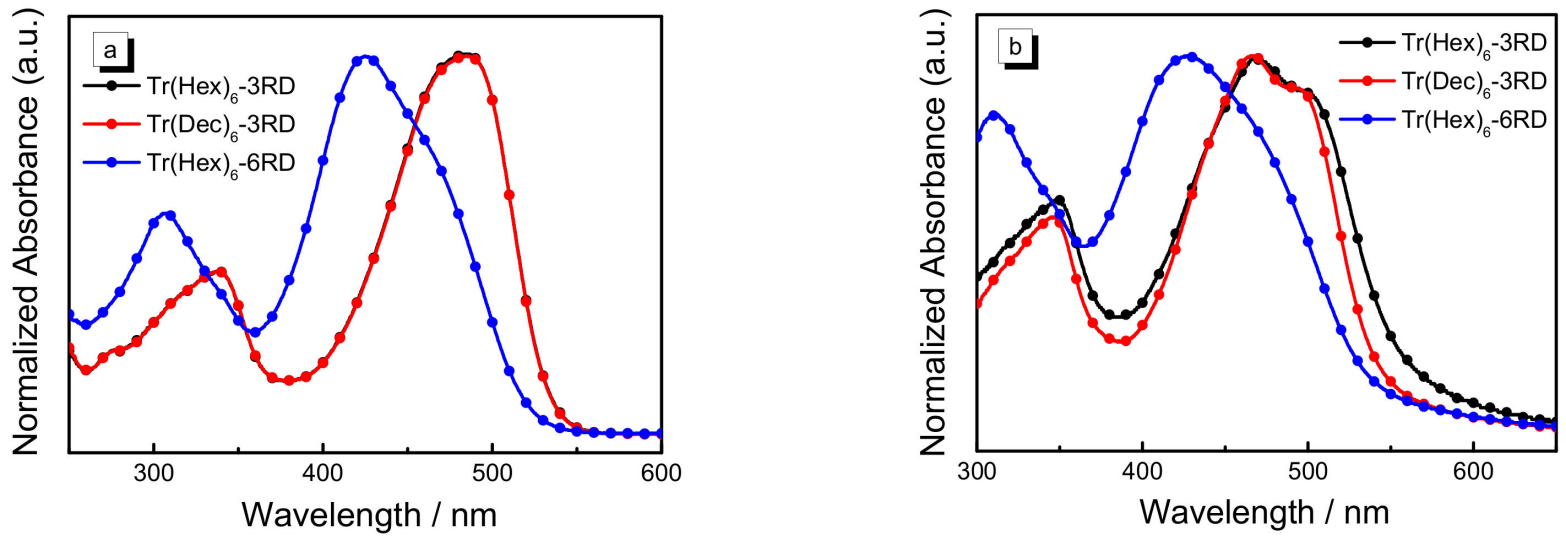
UV-vis absorption spectra of truxene-centered SMAs in chloroform solution (**a**), and as thin films (**b**).

**Optical and electrochemical properties** The solution and thin film UV-vis absorption spectra of three truxene-centered SMAs are displayed in Figure 4. The absorption coefficients of three truxene-centered SMAs are all above 1.8 × 10^5^ cm^−1^ as expected, in particular, Tr(Hex)_6_-3RD achieving the highest one of 2.4 × 10^5^ cm^−1^ (Figure S19). In chloroform solution (Figure 4a), Tr(Hex)_6_-3RD and Tr(Dec)_6_-3RD presented similar absorption at 390~550 nm. Six branched arms of Tr(Hex)_6_-6RD displayed obvious blue-shift compared with three-arms acceptors because of intramolecular twisting. The thin films absorption of these truxene-centered SMAs (Figure 4b) were obviously red-shifted and broad in comparison to those in chloroform solution. The strong intramolecular charge transfer effects in the solid state resulted in the above phenomenon [32]. Obviously, variations in the alkyl chain length on truxene presented modest spectrum alterations, which can be proved by Tr(Hex)_6_-3RD and Tr(Dec)_6_-3RD. In addition, according to the Planck equation (*E*_g_ = 1240/λ_onset_), all these truxene-centered SMAs were calculated to be wide optical band gaps of 2.22 eV for Tr(Hex)_6_-3RD, 2.27 eV for Tr(Dec)_6_-3RD, and 2.33 eV for Tr(Hex)_6_-6RD (Table 1).

Cyclic voltammetry (CV) was used to evaluate the electrochemical levels (Figure S20). The HOMO and LUMO levels were derived from the onset oxidation (*E*_ox_) and reduction (*E*_red_) potentials using the equations of *E*_HOMO_ = −e(*E*_ox_ − *E*_Fc/Fc_+4.8) eV and *E*_LUMO_ =−e (*E*_red_ − *E*_Fc/Fc_ + 4.8) eV, respectively [33]. Here, the *E*_Fc/Fc_ was measured to be 0.36 V. The HOMO levels mainly ranged from −5.8~−6.0 eV, while LUMO levels were around −3.6 eV. The energy levels satisfied the requirement as acceptors. Further, Table 1 summarizes more extensive optical and electrochemical data.

**Figure 5:**
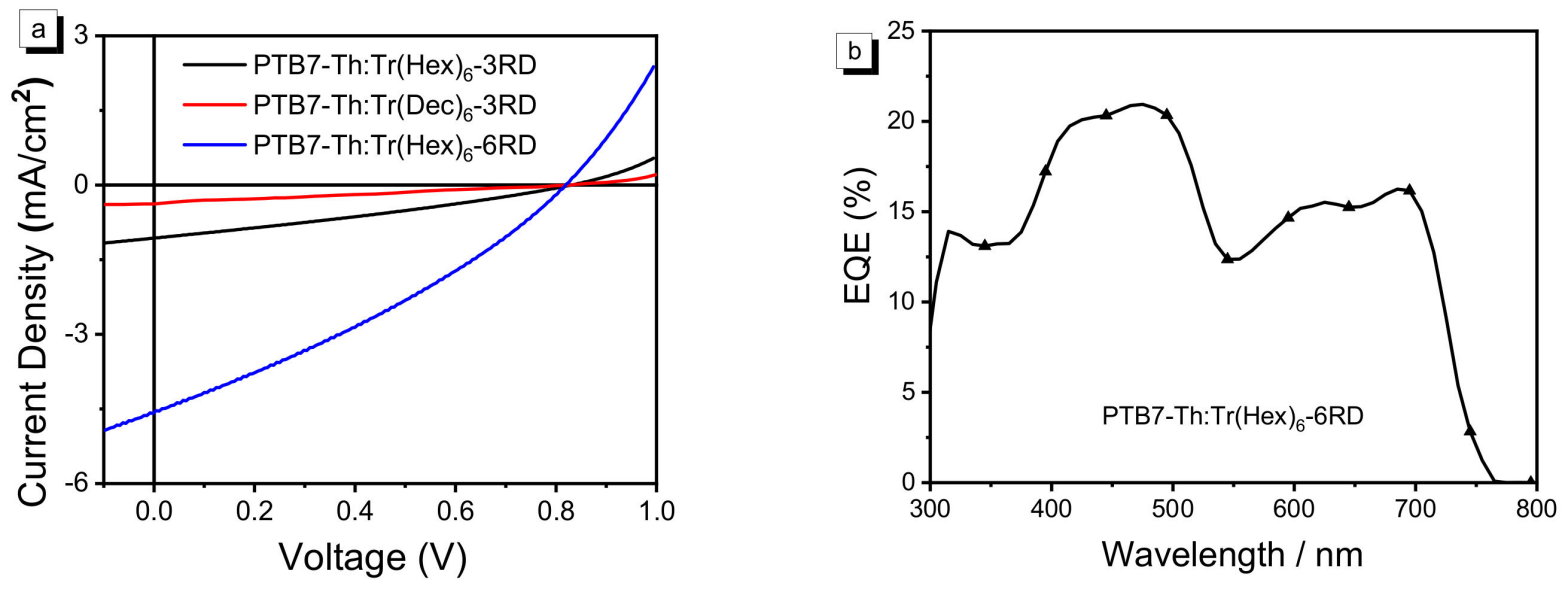
*J–V* characteristics curves of fabricated OSCs devices based on PTB7-Th: truxene-centered SMAs (**a**), and external quantum efficiency (EQE) of PTB7-Th:Tr(Hex)_6_-6RD (**b**).

**Photovoltaic properties** To highlight the potential applications of truxene-centered SMAs in OSCs, OSCs devices with structures of ITO/(PEDOT:PSS)/donors: truxene-centered SMAs/PFN-Br/Ag were fabricated and tested under AM 1.5 G at 100 mW cm^−2^. Given that absorptions of truxene-centered SMAs are primarily located at high energy regions less than 550 nm, PTB7-Th is employed as a donor to satisfy complementing absorption because of the comparatively low-energy region. The photoactive layers were prepared by spin-coating PTB7-Th: truxene-centered SMAs (weight ratio of 1:1) in chloroform solutions. The current density–voltage (*J*–*V*) curves are shown in Figure 5a, and the photovoltaic parameters are listed in Table 2. All the acceptors displayed moderate open-circuit voltage (*V*_oc_) of around 0.8 V; however, the afforded PCEs were not particularly outstanding because of the disappointing short circuit current (*J*_sc_) and fill factor (FF). PTB7-Th: Tr(Hex)_6_-6RD devices reached the maximum PCE of 1.1% with *J*_sc_ of 4.2 mA cm^−2^, *V*_oc_ of 0.82 V, and FF of 31.2%, according to the *J*–*V* curves. The external quantum efficiency (EQE) of PTB7-Th: Tr(Hex)_6_-6RD was illustrated in Figure 5b. The broad EQE ranged from 400 nm to 500 nm was attributed to Tr(Hex)_6_-6RD. Meanwhile, post-annealing of devices was explored (Table S2), and all the devices displayed unsatisfactory *J*_sc_. Furthermore, P3HT: Tr(Hex)_6_-6RD (weight ratio of 1:1)-based devices showed low parameter of open-circuit voltage (*V*_oc_) and PCE (Table S3). PBDB-T: Tr(Hex)_6_-6RD (weight ratio of 1:1) based devices exhibited high *V*_oc_ but low *J*_sc_, resulting in an unfavorable PCE (0.5%, Table S4).

**Figure 6:**
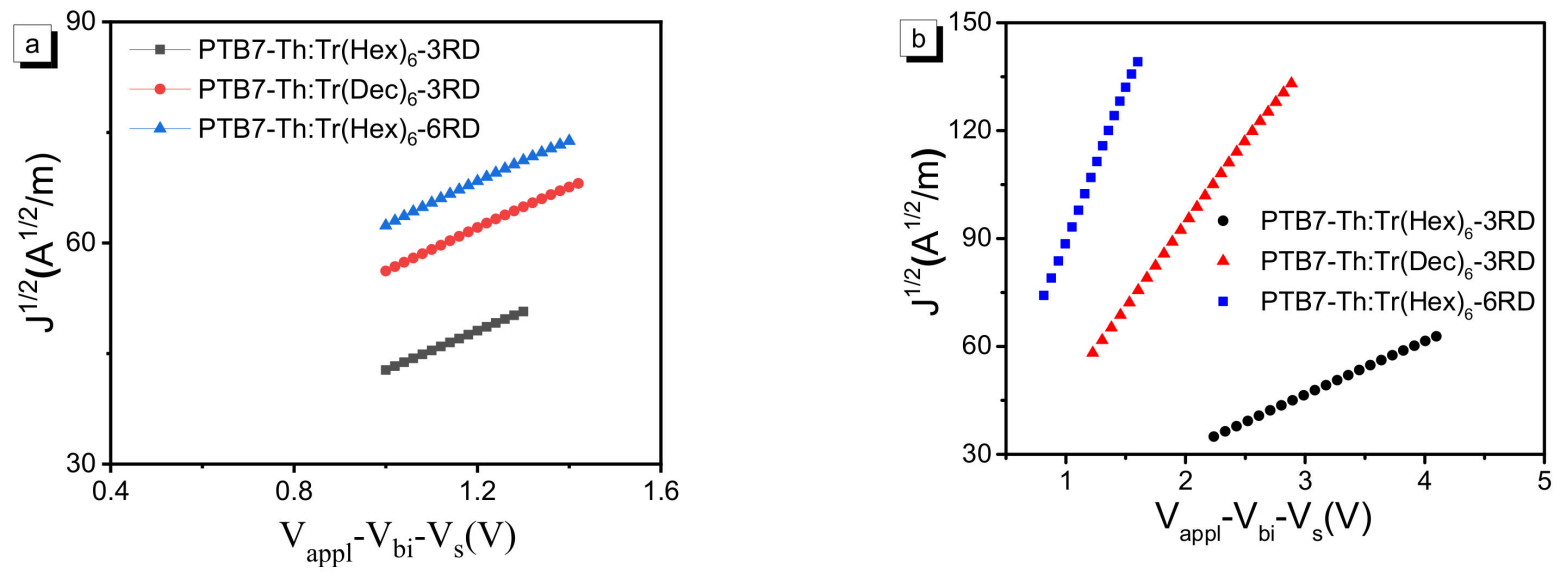
*J*^1/2^–*V* characteristics of PTB7-Th: truxene-centered acceptors blend films in electron-only devices (**a**) and hole-only devices (**b**).

**Carrier Mobility** To investigate the effects of changes in alkyl chain length and branched arm number on the mobility, the electron/hole mobilities of blend films were evaluated by the space-charge-limited current (SCLC) method [34] with a device structure of Ag/PTB7-Th: truxene-centered SMAs/PFN-Br/Ag and ITO/PEDOT:PSS/PTB7-Th: truxene-centered SMAs/MoO_3_/Ag for electron-only devices and hole-only devices, respectively (Figure 6). All of the blend films presented similar electron mobility (*µ*_e_), ranging from 2.48 × 10^−4^ to 2.76 × 10^−4^ cm^2^ V^−1^ s^−^^1^ (Figure 6a). As expected, Tr(Hex)_6_-6RD displayed high electron mobility, which is in good agreement with the FF and PCEs. The hole mobilities (*µ*_h_) for PTB7-Th:Tr(Hex)_6_-3RD, PTB7-Th:Tr(Dec)_6_-3RD, and PTB7-Th: Tr(Hex)_6_-6RD were evaluated to be 6.04 × 10^−4^ cm^2^ V^−1^ s^−1^, 1.54 × 10^−3^ cm^2^ V^−1^ s^−1^, and 5.30 × 10^−3^ cm^2^ V^−1^ s^−1^, on the other hand, and were predicted to be one orders of magnitude greater than those *µ*_e_ (Figure 6b). The charge transport is severely hampered by the low electron mobility and the unbalanced *µ*_e_/*µ*_h_, resulting in more bimolecular recombination and leading to low FF and *J*_sc_.

**Figure 7:**
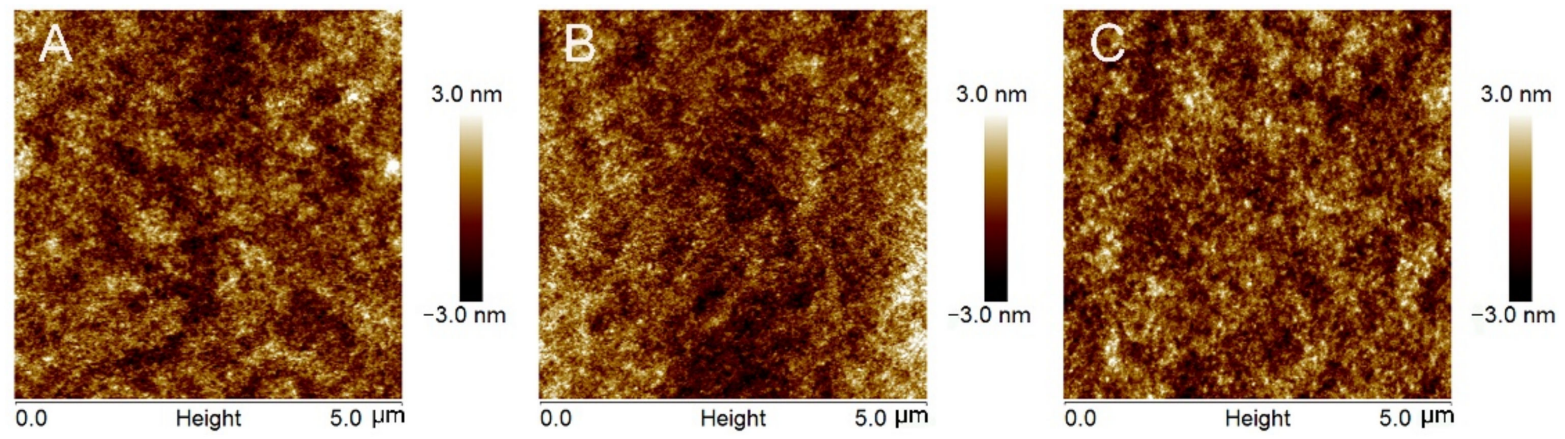
AFM topographic images of PTB7-Th: truxene-centered acceptors blend films, (**A**) PTB7-Th:Tr(Hex)_6_-3RD, (**B**) PTB7-Th:Tr(Dec)_6_-3RD, and (**C**) PTB7-Th:Tr(Hex)_6_-6RD.

**Morphology** The morphologies of PTB7-Th:truxene-centered SMAs blend films were probed via atomic force microscopy (AFM), as shown in Figure 7. All films were prepared by spin-coating process with the same fabrication conditions, which were deposited upon PEDOT:PSS substrate. Tr(Hex)_6_-3RD, Tr(Dec)_6_-3RD, and Tr(Hex)_6_-6RD showed smooth and uniform surface with RMS values of 0.78 nm, 0.65 nm, and 0.86 nm, demonstrating that alkyl chain length and branched arm number changes of these three acceptors have little effect on the morphologies. Six-bladed propeller acceptors of Tr(Hex)_6_-6RD presented noteworthy aggregation and phase separation with PTB7-Th compared with PTB7-Th:Tr(Hex)_6_-3RD and PTB7-Th:Tr(Dec)_6_-3RD. Therefore, exciton separation and carrier collection efficiency are significantly improved, resulting in a high *J*_sc_ and FF.

## 3. Materials and Methods

### Materials

**Compound 1**

1-Indanone (20.0 g, 151 mmol) was dissolved in the mixture solution of acetic acid (120 mL) and concentrated hydrochloric acid (60 mL). The solution was heated to 120 °C and refluxed for 24 h. The hot mixture was poured into 1 L ice water, then sodium carbonate was added slowly and stirred for 1 h. The yellow precipitate was filtered, and washed with water, acetone and dichloromethane to give an off-white powder (Compound **1**) (11 g, 65%); ^1^H NMR (500 MHz, CDCl_3_) δ 7.98 (d, 3H), 7.71 (d, 3H), 7.51 (t, 3H), 7.40 (t, 3H), 4.29 (s, 6H); ^13^C NMR (126 MHz, CDCl_3_) δ 144.21, 142.13, 137.54, 135.71, 127.35, 126.73, 125.56, 122.31, 36.98.

**Compound 2**

This is a lithium–hydrogen exchange reaction. Compound **1** (10 g, 29 mmol) was replaced with dry argon three times. Then anhydrous THF (200 mL) was injected, and the suspension was stirred at −78 °C. n-BuLi (115.2 mL, 2.5 M) was added dropwise and the mixture was kept at −78 °C for 2 h. 1-bromohexene (48.2 g)/1-bromodecane (64.6 g) was injected slowly. The mixture was allowed to warm to room temperature and stirred overnight. After that, the mixture was poured into 1 L of saturated NH4Cl aqueous solution to quench the excess n-BuLi. The water phase was exacted with ethyl acetate, and then the combined organic phase was dried over MgSO4. After the solvent was removed, the crude product was chromatographed on silica gel with hexane to give an off-white powder (Compound **2**) (R = C_6_H_13_, 22 g, 95%), (R = C_10_H_21_, 28 g, 92%). We used this directly in the next step without characterization.

**Compound 3**

A mixture of Compound **2** (R = C_6_H_13_, 2.4 g, 2.8 mmol), anhydrous FeCl3 (5 mg) and chloroform (15 mL) was stirred. Then a solution of bromine (0.5 mL, 10 mmol) in 5 mL of chloroform was added dropwise under stirring at 0 °C, then kept overnight. A Na2SO3 aqueous solution (50 mL) was added to remove excess bromine. The mixture was exacted through DCM three times, and the organic phase was dried over MgSO4. After the solvent was removed, the yellow residue was recrystallized form ethanol to yield an off-white powder (2.65 g, 89%). Compound **3** R = C_6_H_13_: ^1^H NMR (500 MHz, CDCl_3_) δ 8.18 (d, 3H), 7.56 (d, 3H), 7.52 (dd, 3H), 2.87 (m, 6H), 2.04 (m, 6H), 0.93 (m, 36H), 0.63 (m, 18H), 0.46 (m, 14H); ^13^C NMR (126 MHz, CDCl_3_) δ 156.02, 145.04, 139.00, 137.77, 129.51, 126.04, 125.66, 121.18, 56.13, 36.95, 31.59, 29.51, 24.02, 22.40, 14.01; Compound **3** R = C_10_H_21_: ^1^H NMR (500 MHz, CDCl_3_) δ 8.17 (d, 3H), 7.72 (d, 3H), 7.56 (dd, 3H), 2.83 (m, 6H), 2.04 (m, 6H), 0.91 (m, 102H), 0.51 (m, 12H). ^13^C NMR (126 MHz, CDCl_3_) δ 156.22, 145.11, 139.02, 137.55, 129.55, 126.24, 125.67, 121.28, 56.35, 36.98, 32.29, 29.99, 29.97, 29.80, 29.70, 29.61, 24.32, 23.10, 14.28.

**Compound 4**

A mixture of Compound **3** (R = C_6_H_13_, 0.615g, 0.568 mmol), (5-(1,3-dioxolan-2-yl)thiophen-2-yl)trimethylstannane (1.136g, 2.556 mmol), tetrakis(triphenylphosphine)palladium(0) (146 mg) were dissolved in toluene (70 mL) and degassed with argon for 30min. The mixture was refluxed for 48 h, then cooled to room temperature. 1M HCl (90mL) was added into the mixture and stirred overnight. The mixture was exacted with DCM, and the organic phase was dried over MgSO4. After solvent evaporation, the product was purified by short column chromatography on silica gel (eluent, DCM:PE = 1:1) to attain Compound **4** as a yellow solid (408.7 mg, 65%). Compound **4** R = C_6_H_13_: ^1^H NMR (500 MHz, CDCl_3_) δ 9.94 (s, 3H), 8.43 (d, 3H), 7.82 (d, 3H), 7.77 (d, 6H), 7.57 (d, 3H), 2.99 (m, 6H), 2.18 (m, 6H), 0.93 (m, 36H), 0.87 (m, 30H); ^13^C NMR (126 MHz, CDCl_3_) δ 155.34, 155.31, 146.98, 142.93, 142.07, 138.58, 138.24, 138.07, 125.88, 125.58, 124.70, 120.59, 56.71, 37.72, 32.13, 30.10, 24.67, 22.93, 14.55; Compound **4** R = C_6_H_13_: ^1^H NMR (500 MHz, CDCl_3_) δ 9.94 (s, 3H), 8.43 (d, 3H), 7.82 (d, 3H), 7.79 (d, 6H), 7.57 (d, 3H), 2.99 (m, 6H), 2.17 (m, 6H), 1.13 (m, 84H), 0.87 (m, 18H), 0.52 (m, 12H); ^13^C NMR (126 MHz, CDCl_3_) δ 155.05, 155.02, 146.67, 142.60, 141.81, 138.27, 137.90, 131.75, 125.59, 125.22, 124.33, 120.25, 56.38, 37.30, 32.26, 30.08, 29.97, 29.76, 29.67, 29.61, 24.36, 23.00, 14.42.

**Compound 5**

A mixture of Compound **2** (2.00 g, 2.4 mmol), [bis(trifluoroacetoxy)iodo]benzene (4.26 g, 9.9 mmol) and I_2_ (2.11 g, 8.27 mmol) in DCM (100 mL) under argon was stirred at room temperature overnight in the absence of light. Then methanol (50 mL) was poured into the mixture and the expectant precipitation was filtrated and washed with methanol to give Compound **5** (3.03 g, 82%) as a white solid. ^1^H NMR (500 MHz, CDCl_3_) δ 8.80 (s, 3H), 7.95 (s, 3H), 2.70 (m, 6H), 2.06 (m, 6H), 0.99 (m, 36H), 0.69 (m, 18H), 0.52 (m, 12H); ^13^C NMR (126 MHz, CDCl_3_) δ 155.33, 146.04, 141.64, 136.77, 135.04, 133.30, 105.92, 105.16, 56.05, 36.64, 31.54, 29.44, 24.06, 22.45, 14.08.

**Compound 6**

A mixture of Compound **5** (1.6 g, 1 mmol), (5-(1,3-dioxolan-2-yl)thiophen-2-yl)trimethylstannane (4g, 9 mmol), tetrakis(triphenylphosphine)palladium(0) (57.8 mg) was dissolved in DMF (50 mL) and degassed with argon for 30 min. The mixture was refluxed for 48 h, and then cooled to room temperature. 1M HCl (90 mL) was added to the mixture and stirred overnight. The mixture was exacted with DCM, and the organic phase was dried over MgSO4. After solvent evaporation, the product was purified by short column chromatography on silica gel (eluent, DCM:PE = 1:1) to attain Compound **6** as a yellow solid (508.1 mg, 55%). ^1^H NMR (500 MHz, CDCl_3_) δ 9.94 (s, 3H), 9.91 (s, 3H), 8.51 (s, 3H), 7.72 (d, 3H), 7.70 (d, 3H), 7.62 (s, 3H), 2.89 (m, 6H), 2.23 (m, 6H), 0.96 (m, 36H), 0.64 (m, 30H). ^13^C NMR (126 MHz, CDCl_3_) δ 183.26, 183.18, 155.43, 153.08, 152.59, 147.43, 144.42, 144.27, 141.28, 137.78, 137.18, 136.87, 131.69, 131.26, 129.16, 129.02, 127.44, 125.23, 56.69, 37.23, 31.72, 29.65, 24.37, 22.62, 14.29.

**Tr(Hex)6-3RD**

Compound **4** (0.269 g, 0.2287 mmol) and 3-ethylrhodanine (0.553 g, 3.43 mmol) were dissolved in chloroform (15 mL). One drop of piperidine was added and the solution was left to stir at 65 oC overnight. The product was extracted with DCM and dried over MgSO4. After the removal of the solvent, the crude product was chromatographically purified on silica gel (eluting chlorobenzene) to attain Tr(Hex)6-3RD as a brownish red solid (198 mg, 82%). ^1^H NMR (500 MHz, CDCl_3_): δ 8.44 (d, 3H), 7.93 (s, 3H), 7.80 (m, 3H), 7.74 (s, 3H), 7.57 (d, 3H), 7.48 (d, 3H), 4.25 (m, 6H), 3.01 (m, 6H), 2.22 (m, 6H), 1.34 (m, 12H), 0.94 (m, 36H), 0.61 (m, 30H); ^13^C NMR (126 MHz, CDCl_3_): δ 192.55, 167.79, 155.10, 153.39, 146.54, 141.53, 138.34, 137.39, 136.04, 131.79, 125.84, 125.62, 125.10, 124.94, 120.90, 119.79, 56.45, 40.38, 37.48, 31.87, 29.83, 24.41, 22.66, 14.29, 12.73. MS (MALDI-TOF) calcd for C_93_H_111_N_3_O_3_S_9_, 1606.61; found, 1606.88.

**Tr(Dec)6-3RD**

Tr(Dec)6-3RD was synthesized similarly to Tr(Hex)6-3RD, as described in Scheme 1. The product was obtained as a dark purple solid in an 80% yield. ^1^H NMR (500 MHz, CDCl_3_): δ 8.44 (d, 3H), 7.93 (s, 3H), 7.81 (m, 3H), 7.74 (s, 3H), 7.57 (d, 3H), 7.48 (d, 3H), 4.25 (m, 6H), 3.01 (m, 6H), 2.21 (m, 6H), 1.34 (m, 9H), 1.03 (m, 84H), 0.76 (m, 18H), 0.58 (m, 12H); ^13^C NMR (126 MHz, CDCl_3_): δ 192.28, 167.54, 154.87, 153.12, 146.31, 141.30, 138.08, 137.09, 135.77, 131.50, 125.57, 125.37, 124.77, 124.62, 120.62, 119.48, 56.16, 40.12, 37.08, 32.03, 29.82, 29.75, 29.51, 29.43, 29.37, 24.12, 22.75, 14.18, 12.46. MS (MALDI-TOF) calcd for C_117_H_159_N_3_O_3_S_9_, 1942.99; found, 1943.20.

**Tr(Hex)6-6RD**

Tr(Hex)6-6RD was synthesized similarly to Tr(Hex)6-3RD, as described in Scheme 1. The product was obtained as a dark purple solid in 80% yield. ^1^H NMR (500 MHz, CDCl_3_): δ 8.55 (s, 3H), 7.91 (s, 3H), 7.86 (s, 3H), 7.66 (s, 3H), 7.41 (d, 3H), 7.36 (d, 3H), 7.12 (d, 6H), 4.23 (m, 12H), 2.95 (m, 6H), 2.27 (m, 6H), 1.31 (m, 18H), 1.01 (m, 36H), 0.66 (m, 30H); ^13^C NMR (126 MHz, CDCl_3_): δ 192.23, 192.23, 167.49, 167.45, 155.02, 150.68, 150.21, 147.07, 140.87, 139.14, 139.13, 137.70, 134.34, 134.30, 131.37, 130.81, 129.54, 129.40, 127.14, 125.06, 125.01, 124.53, 121.82, 121.64, 56.55, 40.11, 37.19, 31.57, 29.52, 24.19, 22.49, 14.18, 12.46. MS (MALDI-TOF) calcd for C_123_H_132_N_6_O_6_S_18_, 2365.52; found, 2365.73.

**Characterizations**

The ^1^H and ^13^C NMR spectra were tested using a Bruker AV-500 with tetramethylsilane (TMS) as an internal reference. MALDI-TOF-MS was performed by using a BrukerAgilent1290/maXis impact. UV–vis spectra were measured using a HP 8453 spectrophotometer. Thermogravimetric analyses (TGA) were measured using a NETZSCH TG 209 at a heating rate of 10 oC min-1 with a nitrogen flow rate of 20 mL min-1. Cyclic voltammetry data were measured using a CHI600 D electrochemical workstation with Bu4NPF6 (0.1 M) in acetonitrile as the electrolyte, platinum and a saturated calomel electrode as the working and reference electrodes, respectively. The geometry was optimized by Density Functional Theory (DFT) calculations performed at the B3LYP/6-31G(d) level to optimize the ground state geometries of the acceptor molecules using the Gaussian 09. The AFM images were obtained from a NanoMan VS microscope with a tapping mode.

OSCs devices are ITO/(PEDOT:PSS)/donors: truxene-centered SMAs/PFN-Br/Ag, PEDOT:PSS represents poly(3,4-ethylenedioxythiophene) –poly(styrenesulfonate) and PTB7-Th represents poly[[2,6-4,8-di(5-ethylhexylthienyl)benzo[1,2-b;3,3-b]-dithiophene][3-fluoro-2[(2-ethylhexyl)carbonyl]thieno[3,4-b]-thiophenediyl). The charge transport was acquired by single-carrier devices with a device structure of Ag/PTB7-Th (truxene-centered SMAs/PFN-Br/Ag for electron only devices) and ITO/PEDOT:PSS/(PTB7-Th) (truxene-centered SMAs/MoO3/Ag for hole only devices).

## 4. Conclusions

In summary, a series of high absorption coefficient truxene-centered small molecular acceptors (SMAs) with distinct molecular topologies were synthesized and explored for high performance organic solar cells. The multifarious structure modifications (multi-branched arms) and side chain engineering (hexyl to decyl chains) of SMAs were also evaluated from thermal, optical, electrochemical properties, mobility, morphologies, and device performance. In combination with a narrow band-gap donor PTB7-Th, six branched arms with hexyl chain of Tr(Hex)_6_-6RD achieved the best PCEs of 1.1% due to the high *µ*_e_ and *µ*_h_ and favorable morphology.

## Figures and Tables

**Table 1:** Optical and electrochemical properties of truxene-centered SMAs.

Acceptors	λ_max_^sol^ (nm)	λ_max_^film^ (nm)	*E*_g_^opt a^ (eV)	*E*_ox_ (V)	*E*_red_ (V)	*E*_HOMO_ ^b^ (eV)	*E*_LUMO_ ^c^ (eV)	*E*_g_^CV d^ (eV)
Tr(Hex)_6_-3RD	481	468	2.22	1.35	−0.85	−5.78	−3.58	2.20
Tr(Dec)_6_-3RD	481	468	2.27	1.41	−0.83	−5.84	−3.60	2.24
Tr(Hex)_6_-6RD	425	428	2.33	1.54	−0.83	−5.97	−3.60	2.37

^a^ Calculated from *E*_g_^opt^ = (1240/λ_onset_^film^) eV; ^b^ Calculated from *E*_HOMO_ = −e(*E*_ox_ − *E*_Fc/Fc_ + 4.8) eV; ^c^ Calculated from *E*_LUMO_ = −e(*E*_red_ − *E*_Fc/Fc_ + 4.8) eV; ^d^ Calculated from *E*_g_^CV^ = *E*_LUMO_ − *E*_HOMO_.

**Table 2:** Photovoltaic parameters of PTB7-Th: truxene-centered SMAs devices.

PTB7-Th	*V*_oc_ (V)	*J*_sc_ (mA cm^−2^)	FF (%)	PCE (%)
Tr(Hex)_6_-3RD	0.83	1.0	29.2	0.3
Tr(Dec)_6_-3RD	0.81	0.4	25.7	0.1
Tr(Hex)_6_-6RD	0.82	4.2	31.2	1.1

## Data Availability

The data are available in this publication and Supplementary Materials.

## Supplementary Materials

The following supporting information can be downloaded at: https://www.mdpi.com/article/10.3390/ijms231810402/s1.
