# Supplementary material for: Truxene-Centered Electron Acceptors for Non-Fullerene Solar Cells: Alkyl Chain and Branched Arm Engineering
Source: Int J Mol Sci. 2022 Sep 8;23(18):10402. doi: 10.3390/ijms231810402 (PMC9499097; doi:10.3390/ijms231810402)
Supplement: Supplementary file 1 [file ijms-23-10402-s001.zip › ijms-1876028-supplementary.pdf]

Truxene-centered Electron Acceptors for Non-Fullerene Solar Cells Alkyl Chain and Branched Arm Engineering

Kaiwen Lin[a,b]*, Wenhao Du[a], Shuqi Shen[a], Haoshen Liang[a], Xiaobin Zhang[a], Manjun Xiao[c] and Yuehui Wang[a]

a Department of Materials and Food, University of Electronic Science and Technology of China Zhongshan Institute, Zhongshan 528402, China
b School of Optoelectronic Science and Engineering, University of Electronic Science and Technology of China, Chengdu 610054, China
c College of Chemistry, Key Lab of Environment-Friendly Chemistry and Application (Ministry of Education), Xiangtan University, Xiangtan 411105, China
* Correspondence: kevinlin1990@zsc.edu.cn Phone: (+)86-791-88537967. Fax: (+)86-791-83823320.

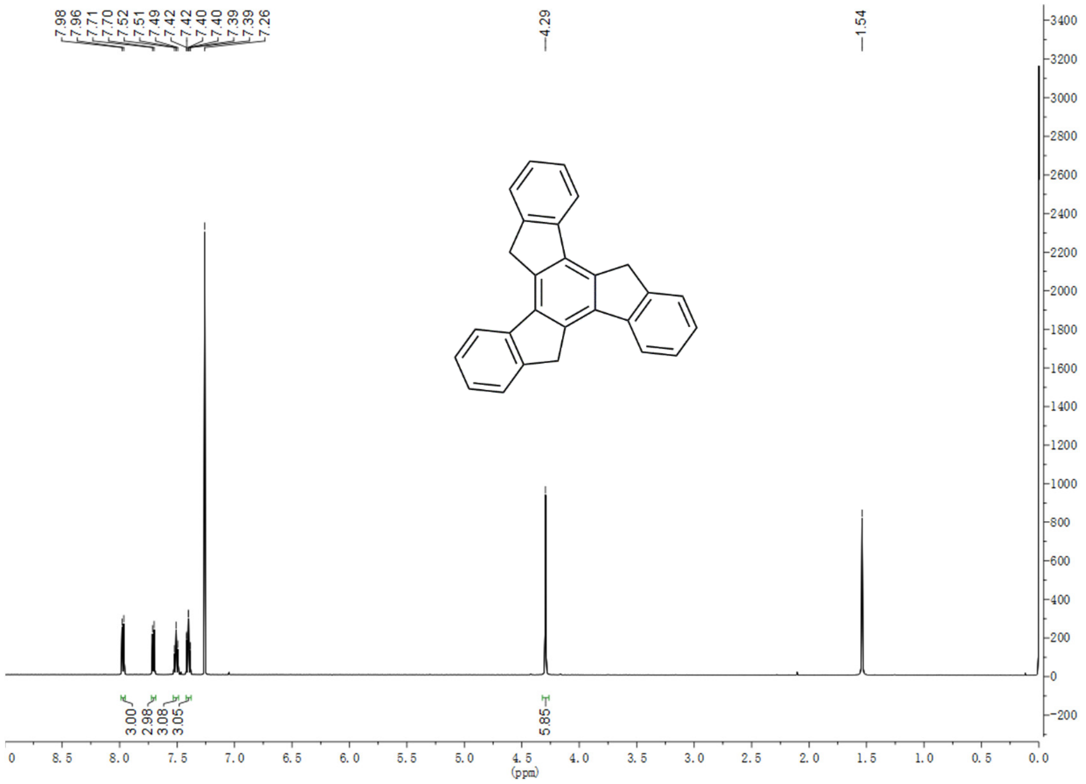

Figure S1. ^{1}H NMR spectrum of compound **1.**

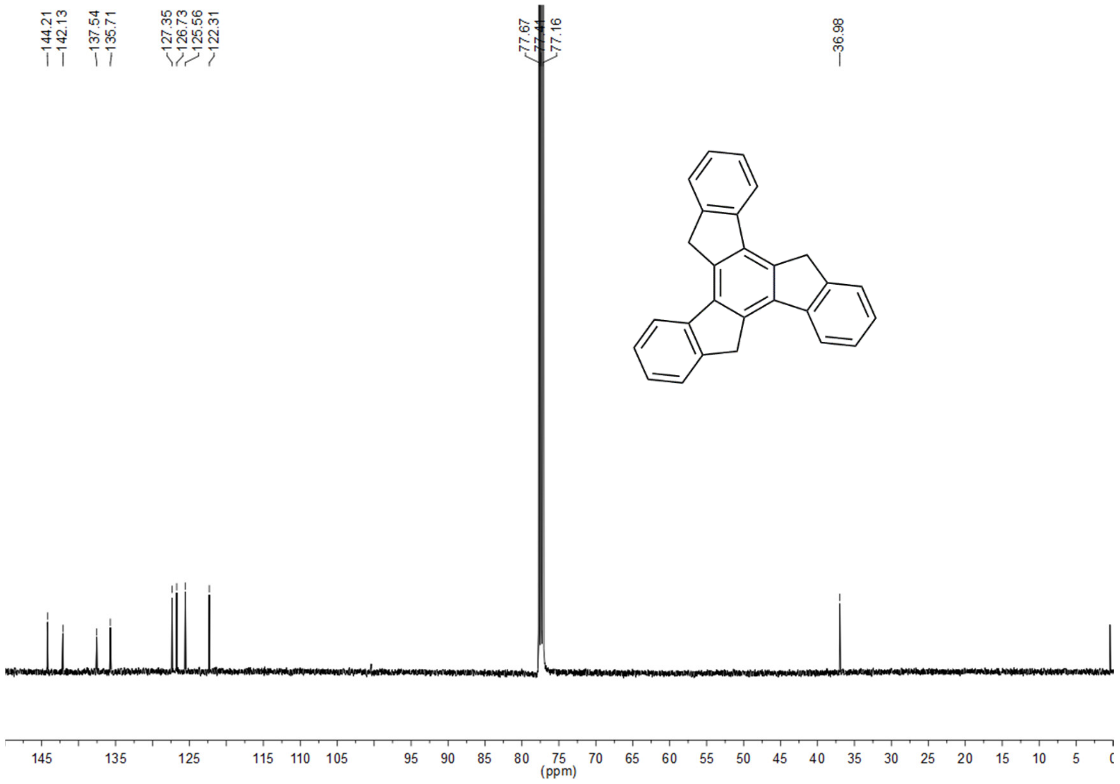

Figure S2. ^{13}C NMR spectrum of compound **1.**

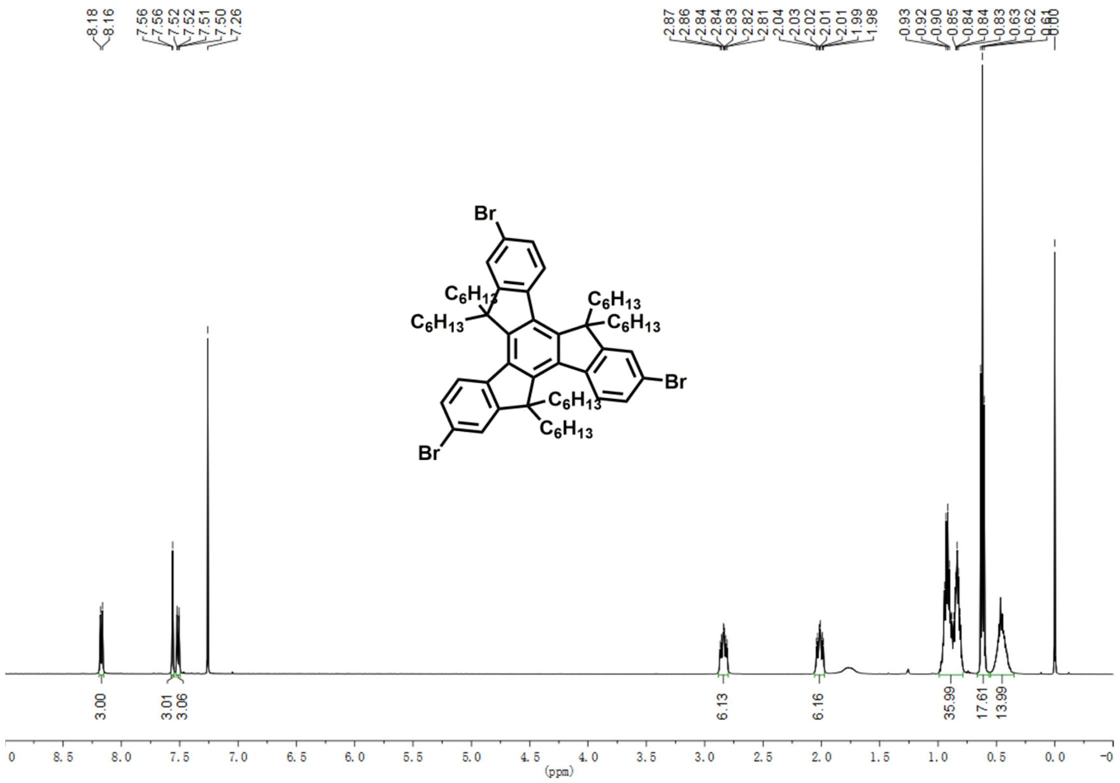

Figure S3. ^{1}H NMR spectrum of compound **3a.**

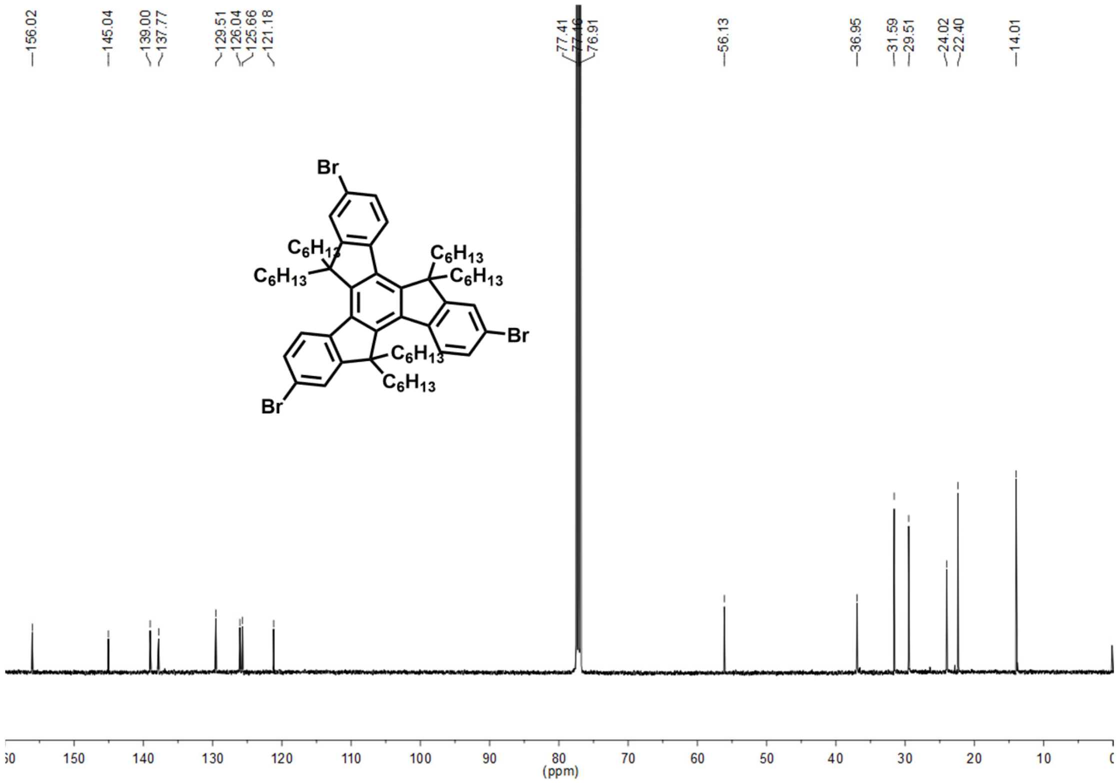

Figure S4. ^{13}C NMR spectrum of compound **3a.**

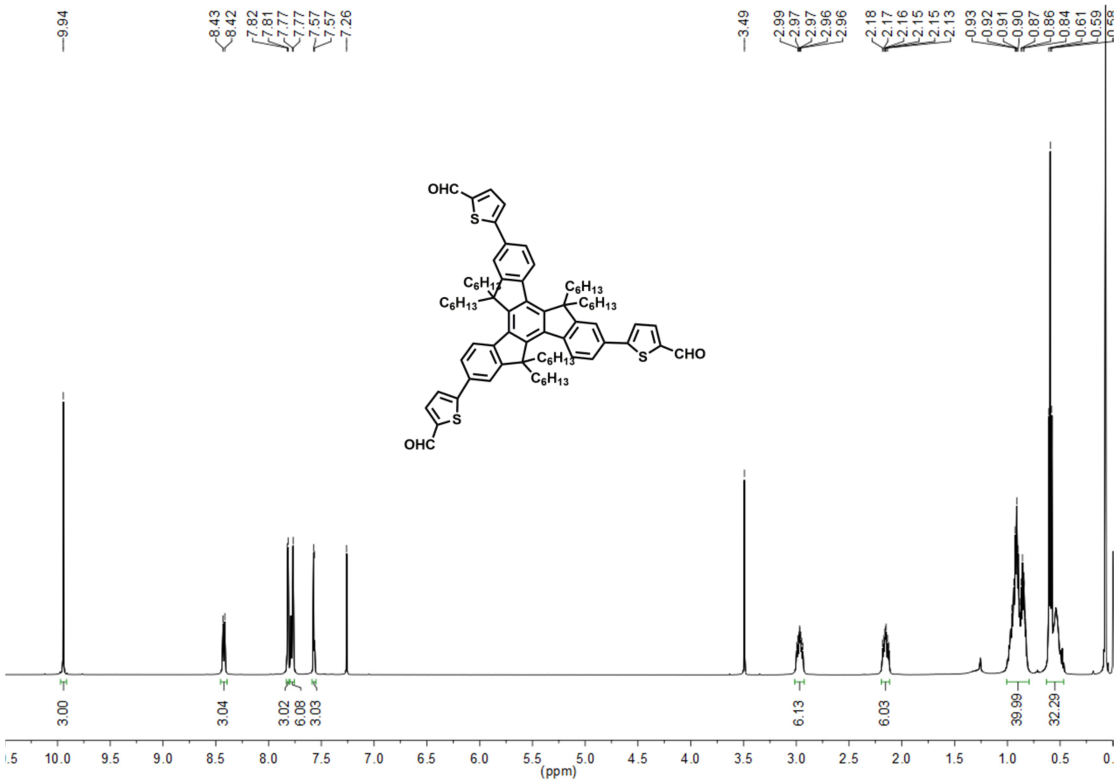

Figure S5 ^{1}H NMR spectrum of compound **4a**

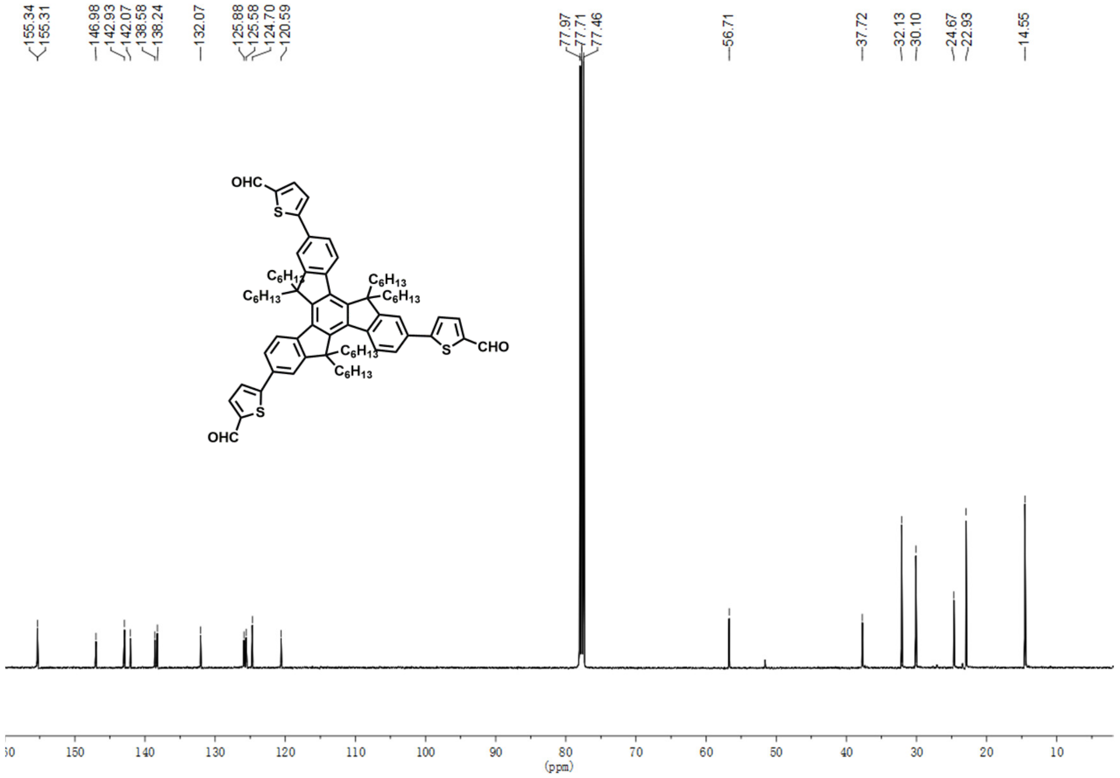

Figure S**6** ^{13}C NMR spectrum of compound **4a**

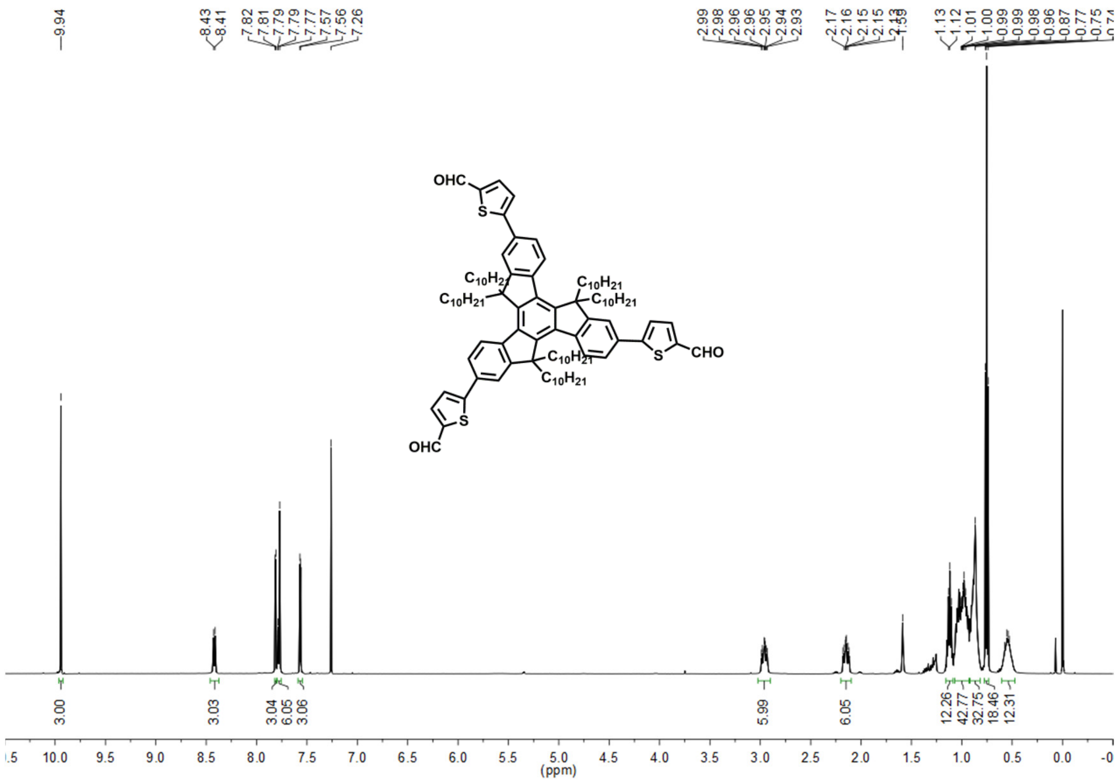

Figure S7 ^{1}H NMR spectrum of compound **4b**

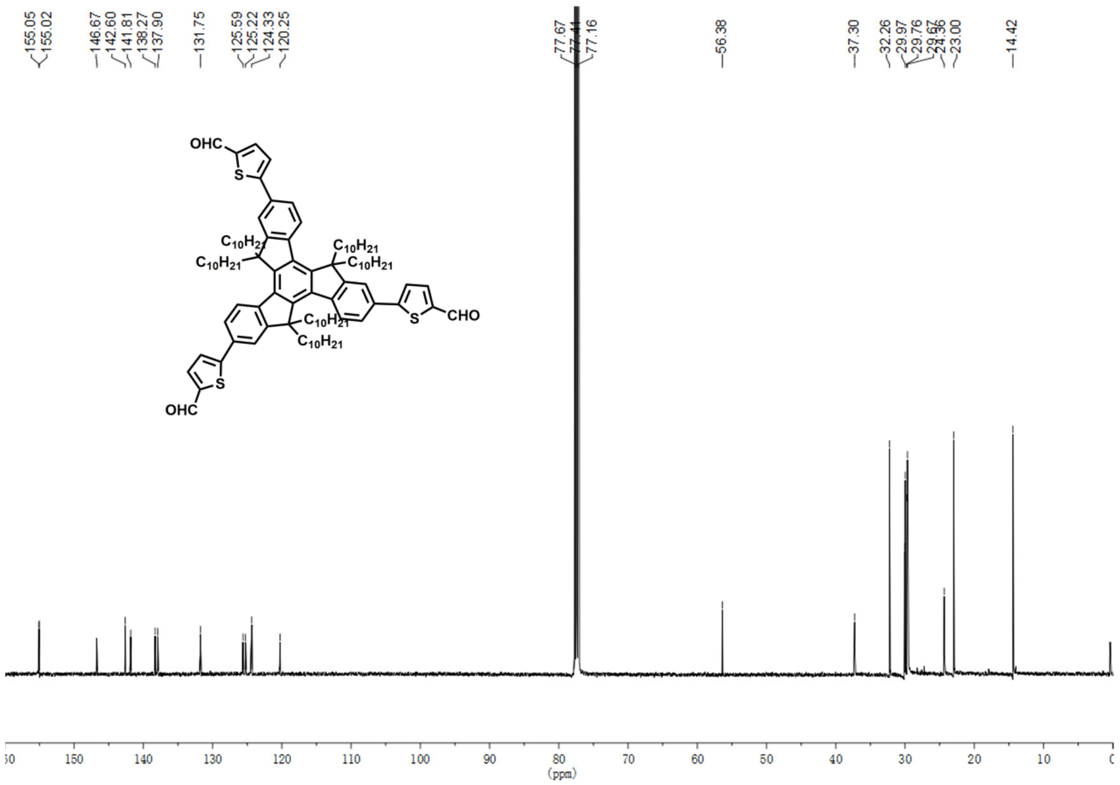

Figure S**8** ^{13}C NMR spectrum of compound **4b**

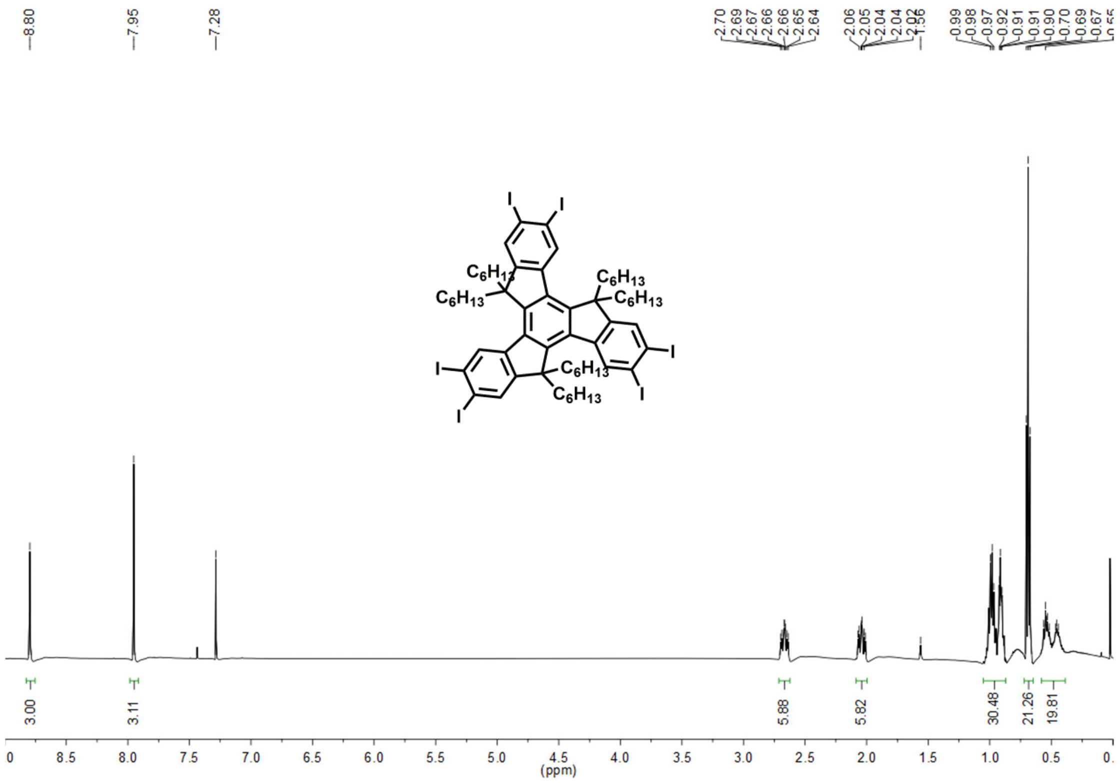

Figure S9 ^{1}H NMR spectrum of compound **5**

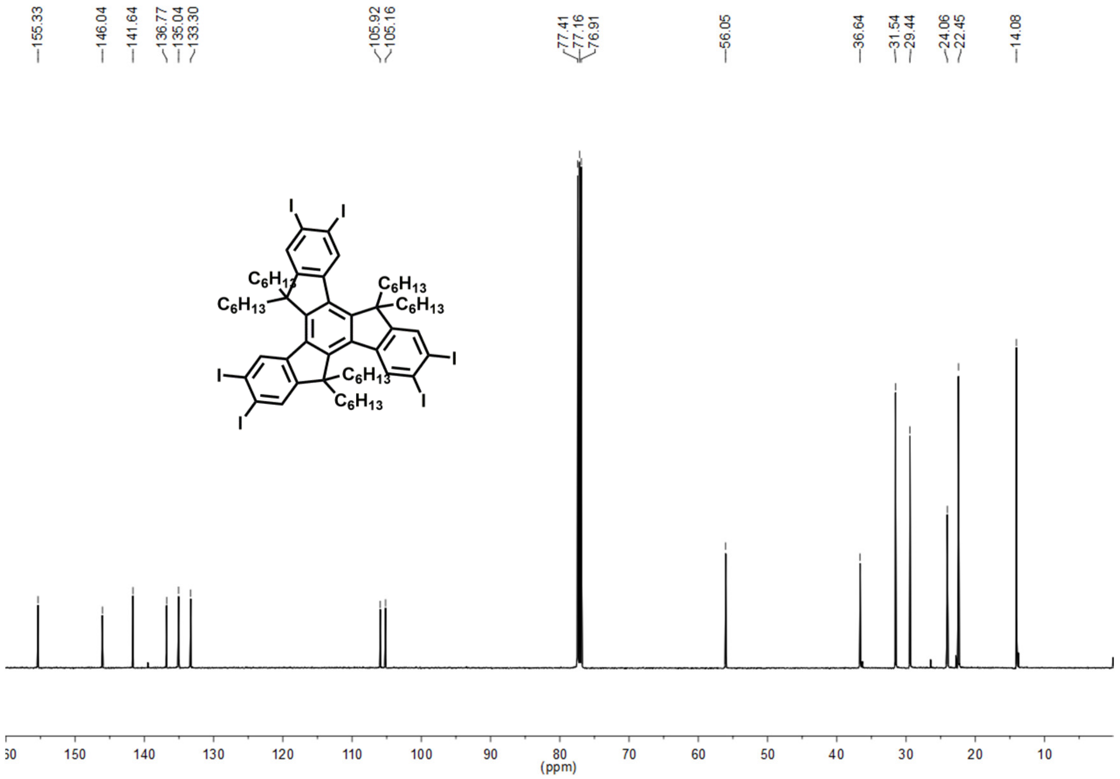

Figure S**10** ^{13}C NMR spectrum of compound **5**

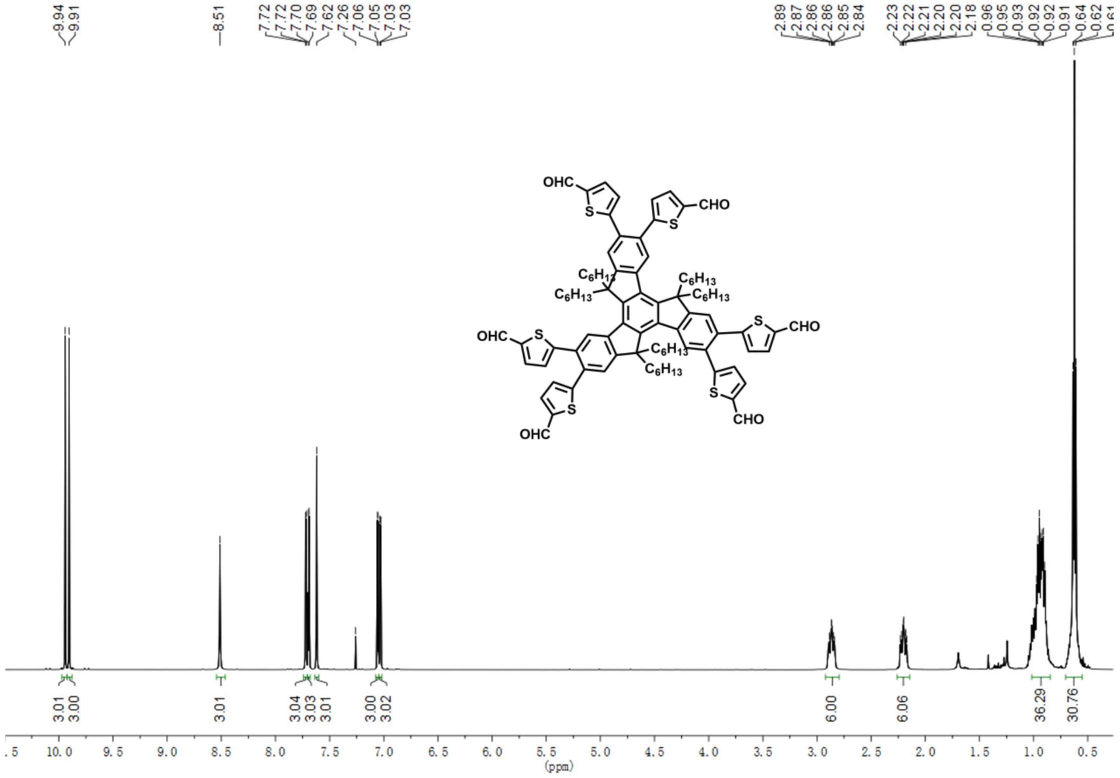

Figure S11 ^{1}H NMR spectrum of compound **6**

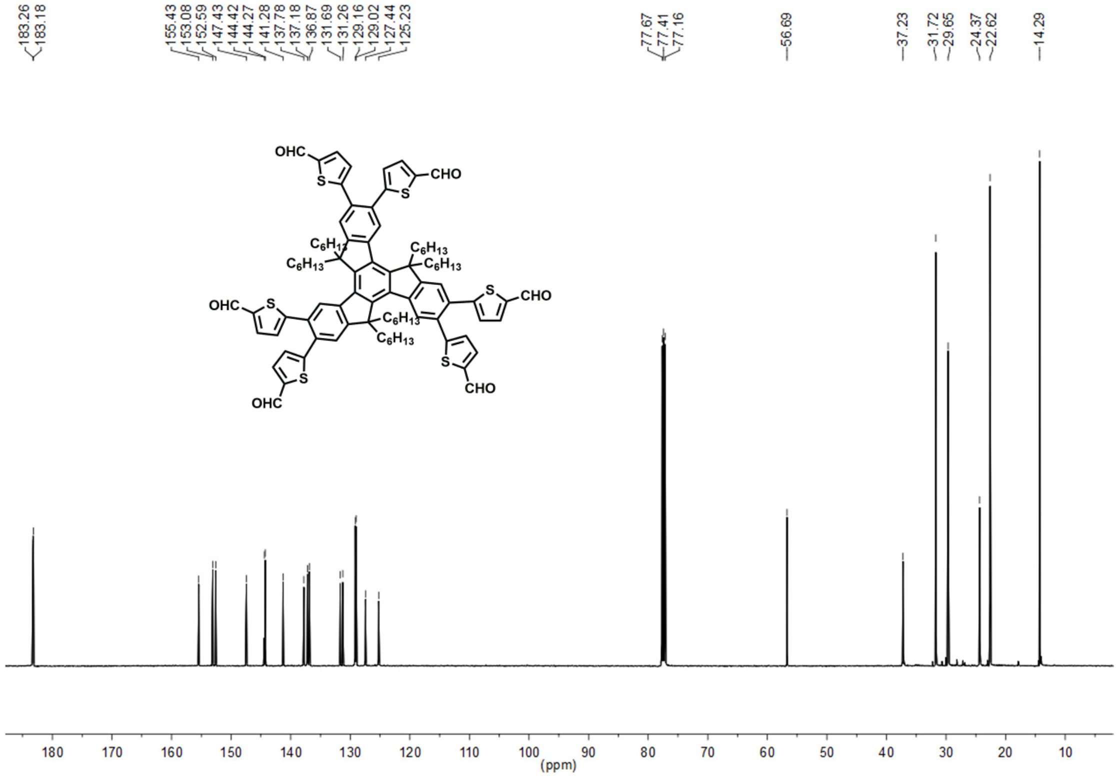

Figure S**12** ^{13}C NMR spectrum of compound **6**

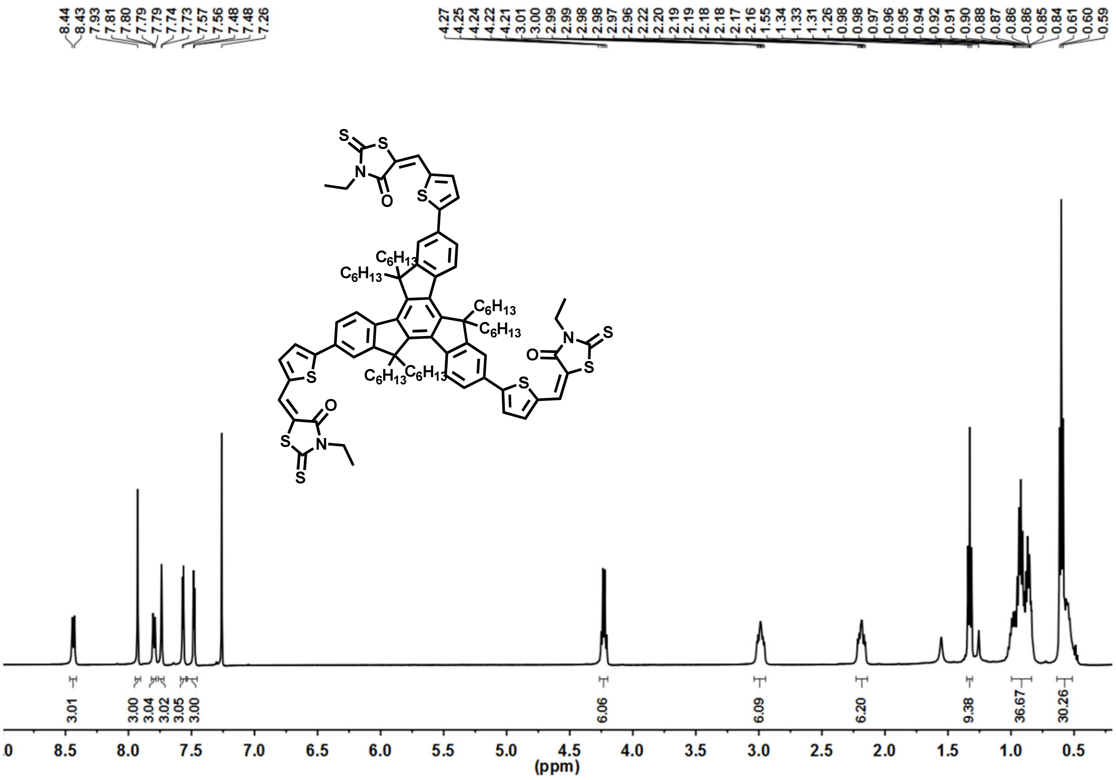

Figure S13 ^{1}H NMR spectrum of Tr(Hex)$_6$-3RD

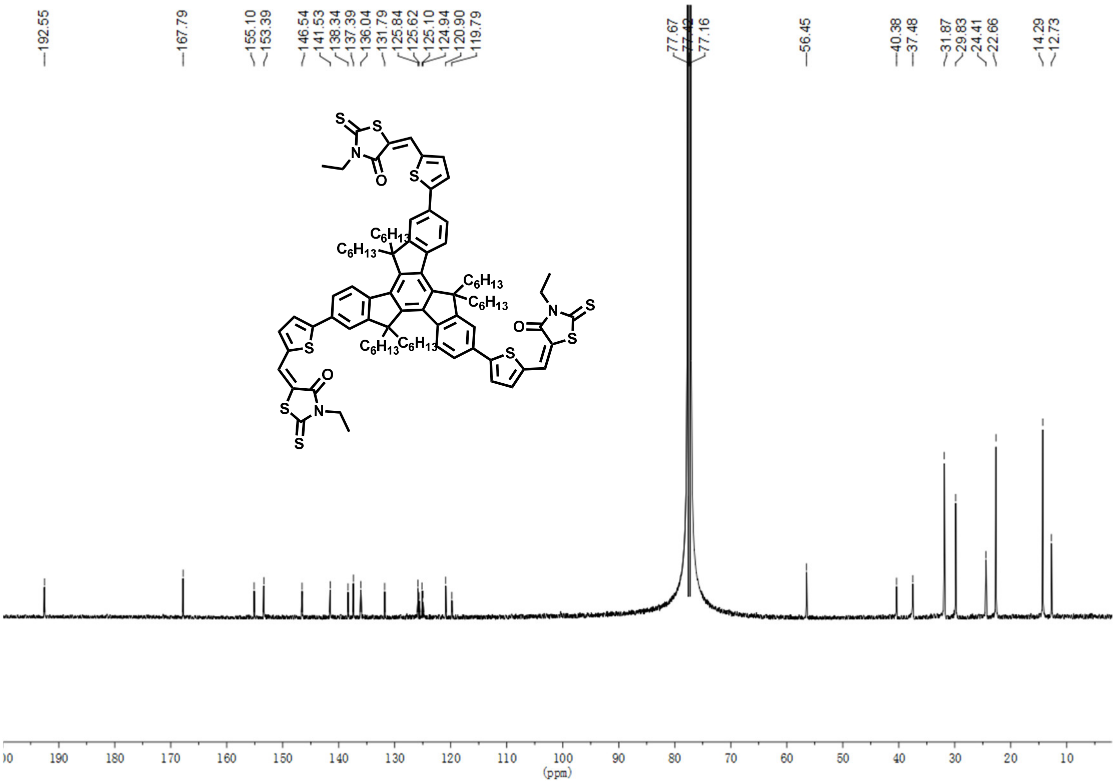

Figure S14 ^{13}C NMR spectrum of Tr(Hex)$_6$-3RD

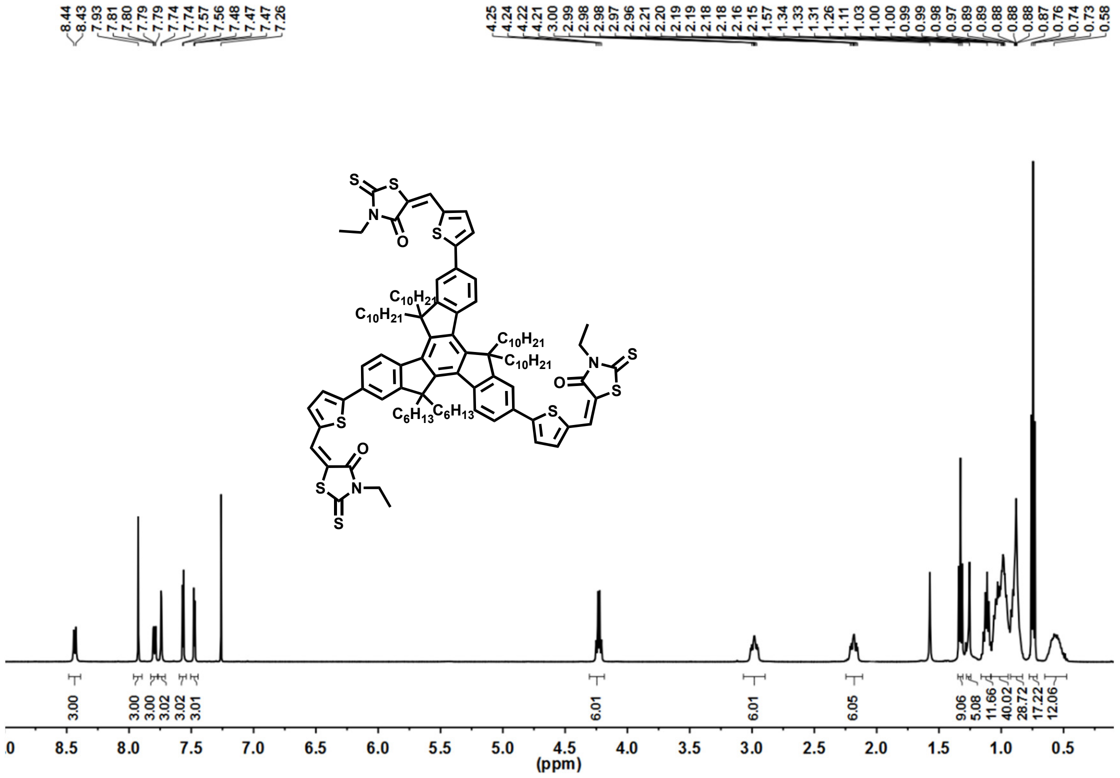

Figure S15 ^{1}H NMR spectrum of Tr(Dec)$_6$-3RD

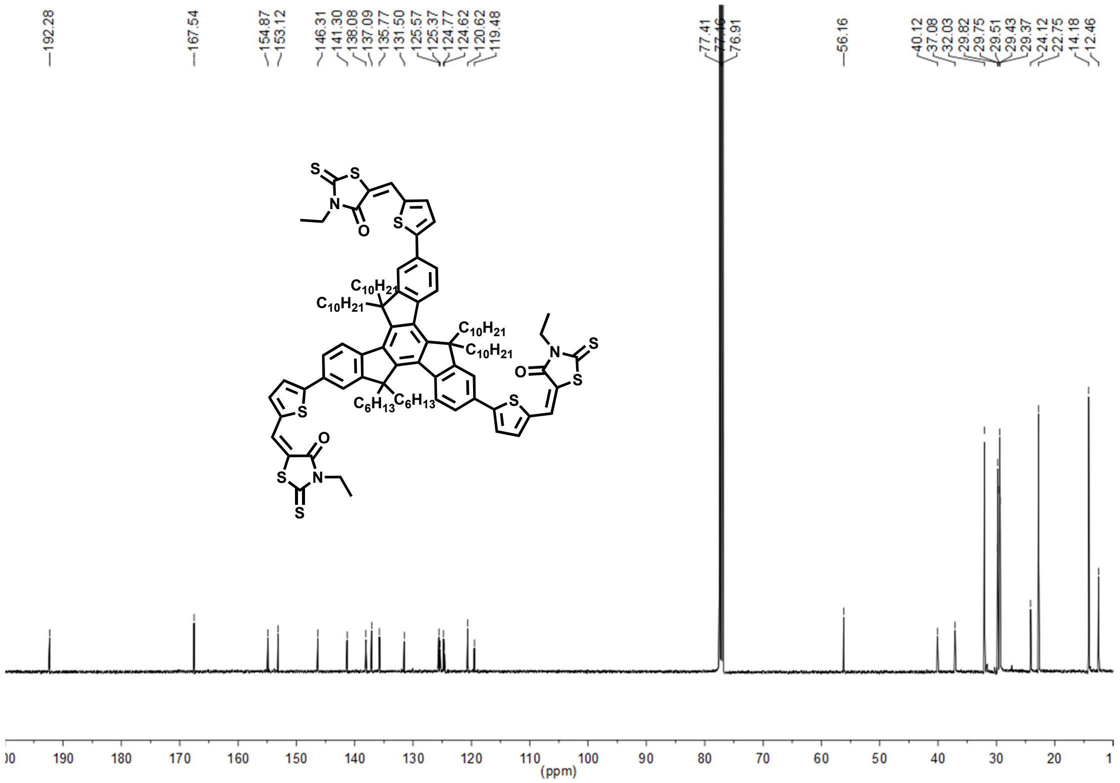

Figure S16 ^{13}C NMR spectrum of Tr(Dec)$_6$-3RD

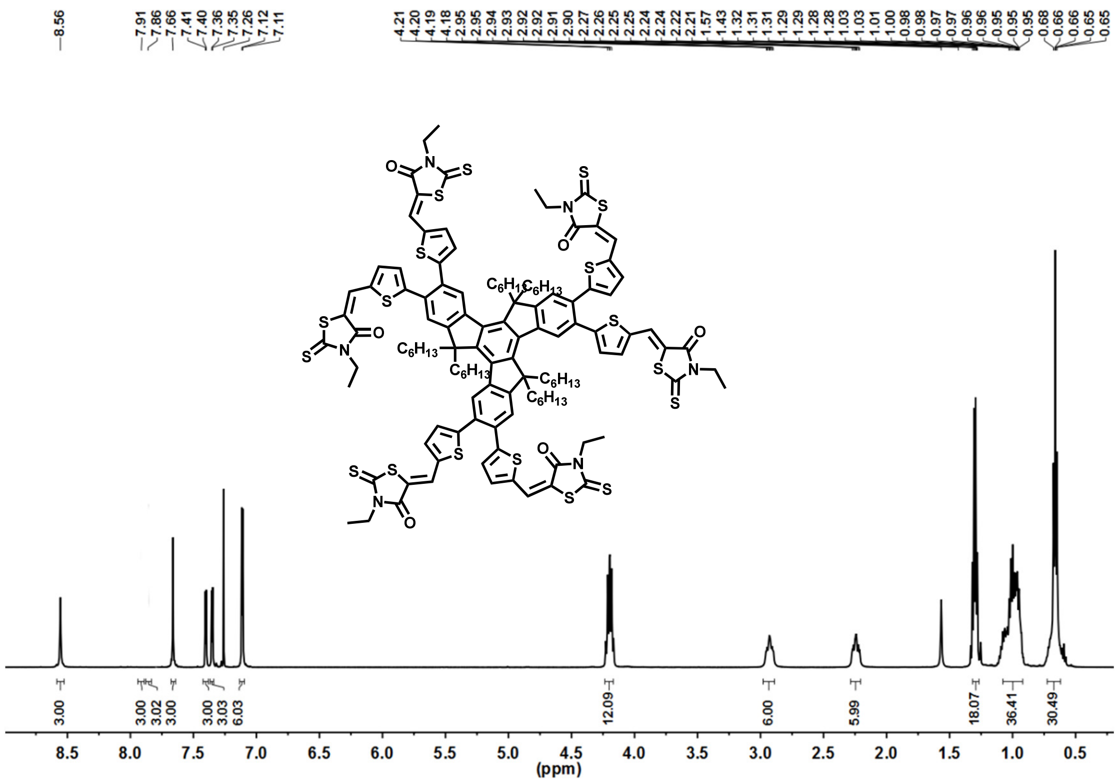

Figure S17 ^{1}H NMR spectrum of Tr(Hex)$_6$-6RD

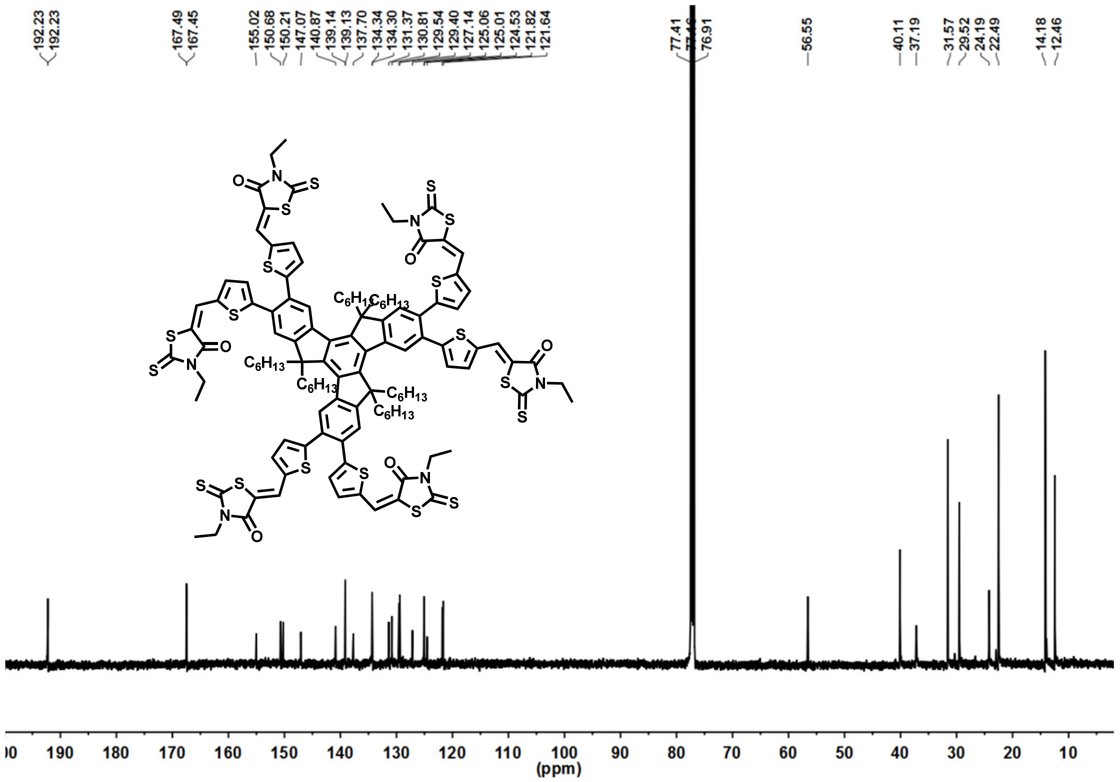

Figure S18 ^{13}C NMR spectrum of Tr(Hex)$_6$-6RD

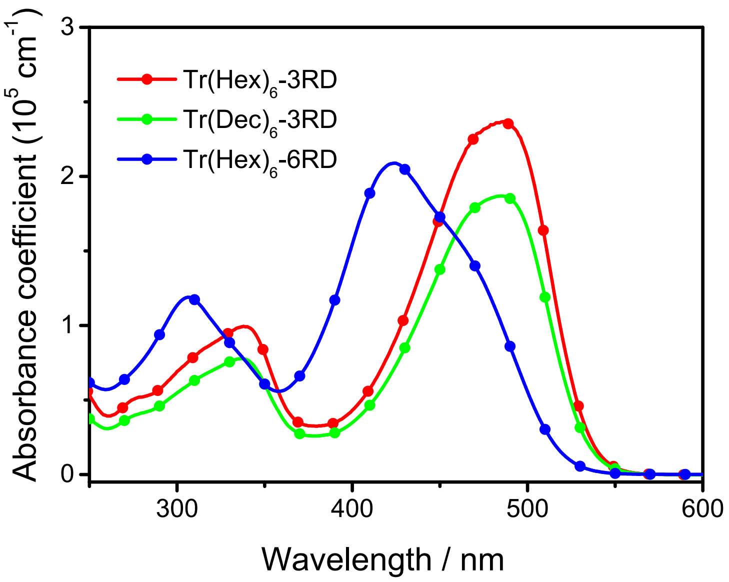

Figure S19 UV-vis absorption spectra of truxene-centered acceptors in chloroform solution

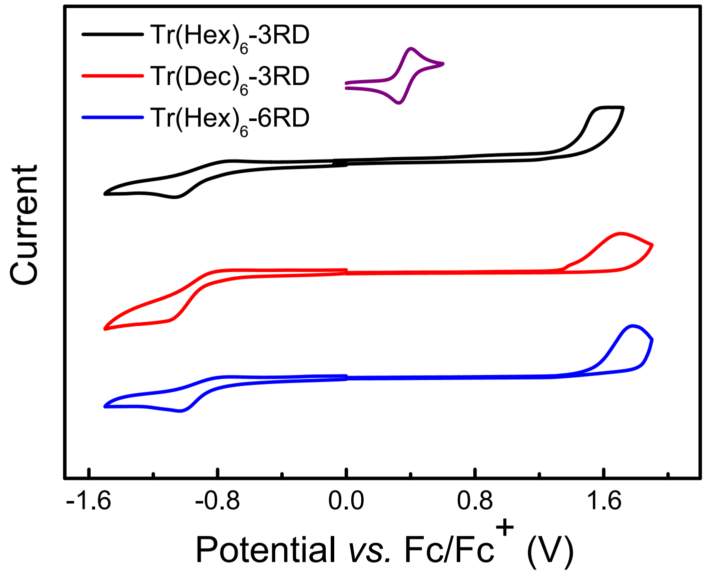

Figure S20 Cyclic voltammograms of truxene-centered acceptors

Table S1. Determined solubilites and thermal parameters of truxene-centered acceptors in chloroform

Acceptors	Solubility (mg/mL)	T_d (°C)	T_m (°C)	T_c (°C)
Tr(Hex)$_6$-3RD	80	410	259	203/190
Tr(Dec)$_6$-3RD	130	404	178	--
Tr(Hex)$_6$-6RD	180	410	--	--

Table S2. Photovoltaic parameters of PTB7-Th: truxene-centered acceptors (weight ratio of 1:1) devices

PTB7-Th: Acceptors	Post-annealing	V_{oc} (V)	J_{sc} (mA cm^{-2})	FF (%)	PCE (%)
PTB7-Th: Tr(Hex)$_6$-3RD	60 °C	0.85	0.2	32.9	0.1
	100 °C	0.84	0.2	31.7	0.1
PTB7-Th: Tr(Dec)$_6$-3RD	60 °C	0.84	0.2	33.5	0.1
	100 °C	0.85	0.2	32.6	0.1
PTB7-Th: Tr(Hex)$_6$-6RD	60 °C	0.84	0.2	31.1	0.1
	100 °C	0.85	0.2	30.1	0.1

Table S3. Photovoltaic parameters of P3HT: $Tr(Hex)_6$-6RD (weight ratio of 1:1) devices

Rotational speed	V_{oc} (V)	J_{sc} (mA cm^{-2})	FF (%)	PCE (%)
1200	0.65	0.6	28.4	0.1
1400	0.59	0.6	29.7	0.1
1600	0.55	0.6	27.7	0.1

Table S4. Photovoltaic parameters of PBDB-T: $Tr(Hex)_6$-6RD (weight ratio of 1:1) devices

Rotational speed	V_{oc} (V)	J_{sc} (mA cm^{-2})	FF (%)	PCE (%)
1200	1.06	1.2	27.8	0.4
1400	1.09	1.2	28.6	0.4
1600	1.09	1.2	28.7	0.4
